# Foraging ecology drives social information reliance in an avian eavesdropping community

**DOI:** 10.1002/ece3.5561

**Published:** 2019-09-14

**Authors:** Harrison H. Jones, Kathryn E. Sieving

**Affiliations:** ^1^ Department of Wildlife Ecology and Conservation University of Florida Gainesville FL USA; ^2^ Department of Biology University of Florida Gainesville FL USA

**Keywords:** antipredator behavior, call relevance, eavesdropping, foraging ecology, Paridae, social information, winter bird community

## Abstract

Vertebrates obtain social information about predation risk by eavesdropping on the alarm calls of sympatric species. In the Holarctic, birds in the family Paridae function as sentinel species; however, factors shaping eavesdroppers' reliance on their alarm calls are unknown. We compared three hypothesized drivers of eavesdropper reliance: (a) foraging ecology, (b) degree of sociality, and (c) call relevance (caller‐to‐eavesdropper body‐size difference). In a rigorous causal‐comparative design, we presented Tufted Titmouse (*Baeolophus bicolor*) alarm calls to 242 individuals of 31 ecologically diverse bird species in Florida forests and recorded presence/absence and type (diving for cover or freezing in place) of response. Playback response was near universal, as individuals responded to 87% of presentations (*N* = 211). As an exception to this trend, the sit‐and‐wait flycatcher Eastern Phoebe (*Sayornis phoebe*) represented 48% of the nonresponses. We tested 12 predictor variables representing measures relevant to the three hypothesized drivers, distance to playback speaker, and vulnerability at time of playback (eavesdropper's microhabitat when alarm call is detected). Using model‐averaged generalized linear models, we determined that foraging ecology best predicted playback response, with aerial foragers responding less often. Foraging ecology (distance from trunk) and microhabitat occupied during playback (distance to escape cover) best predicted escape behavior type. We encountered a sparsity of sit‐and‐wait flycatchers (3 spp.), yet their contrasting responses relative to other foraging behaviors clearly identified foraging ecology as a driver of species‐specific antipredator escape behavior. Our findings align well with known links between the exceptional visual acuity and other phenotypic traits of flycatchers that allow them to rely more heavily on personal rather than social information while foraging. Our results suggest that foraging ecology drives species‐specific antipredator behavior based on the availability and type of escape cover.

## Graphical abstract

Reliance on social information about predators is pervasive in nature, yet the relative importance of ecological factors determining reliance on it is unknown. Using playback of alarm calls from a sentinel species presented to an entire subtropical bird community, we compared three competing hypotheses: foraging ecology, sociality, and call relevance. We found that nonreliance on social information was best predicted by foraging ecology, with sit‐and‐wait flycatchers rarely responding.

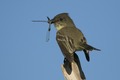

## 1 INTRODUCTION

### 1.1 Animal information networks

Vertebrates must constantly seek information about their surroundings to reduce uncertainty and make adaptive behavioral choices (Dall, Giraldeau, Olsson, McNamara, & Stephens, 2005; Danchin, Giraldeau, Valone, & Wagner, 2004; Schmidt, Dall, & van Gils, 2010; Seppänen, Forsman, Mönkkönen, & Thomson, 2007). Information from direct interaction with the environment (personal information) is combined with cues or signals obtained from other individuals (social information; Danchin et al., 2004) of same or different species. Social information often comes from heterospecifics (Goodale, Beauchamp, Magrath, Nieh, & Ruxton, 2010) because ecologically similar species that share predators and diet items are both collectively more abundant than conspecifics and better able to detect relevant threats and opportunities (Goodale & Kotagama, 2005a). Information exchange between individuals occurs primarily via interceptive eavesdropping in vertebrates (Magrath, Haff, Fallow, & Radford, 2015) and typically on alarm calls—a class of vocalizations in birds and mammals that are used to warn conspecifics about predators (Caro, 2005). Alarm calls encode information about predator type (Seyfarth, Cheney, & Marler, 1980; Suzuki, 2012) and relative risk (Leavesley & Magrath, 2005; Templeton, Greene, & Davis, 2005); therefore, eavesdroppers benefit by adopting appropriate antipredator behaviors.

The production and consumption of social information in a community constitutes an “information network” (Goodale et al., 2010; Schmidt et al., 2010; Seppänen et al., 2007). Information networks are often asymmetrical in nature: A vocally complex “information‐producing” species serves a diverse audience of heterospecific eavesdroppers (Contreras & Sieving, 2011; Goodale et al., 2010; Schmidt et al., 2010). Knowing which species eavesdrop, and the relative value of the social information provided to different eavesdroppers in a network, is fundamental to defining the role of information sharing at the community level (Goodale & Kotagama, 2008; Magrath, Pitcher, & Gardner, 2009; Martínez & Zenil, 2012). Where eavesdropping species rely on antipredator cues provided by heterospecifics, social information provides a mechanism for large‐scale facilitation (Contreras & Sieving, 2011; Hetrick & Sieving, 2012; Szymkowiack, 2013) and an important benefit from participation in mixed‐species foraging groups (Martínez, Parra, Muellerklein, & Vredenburg, 2018; Pagani‐Núñez et al., 2018). There is therefore a need to quantify how reliance on social information may vary among species.

### 1.2 Factors determining the value of social information to eavesdroppers

Species traits and environmental factors both influence whether a species can personally collect all necessary information or must rely on social information (Parejo & Aviles, 2016; Seppänen et al., 2007). Some species are better at detecting threats by virtue of their foraging ecology (Goodale et al., 2010): Species that forage on substrates (substrate‐based foragers) suffer from visual occlusion by foliage, while aerially foraging species (salliers) can scan for prey items and predators simultaneously. Among forest birds, substrate‐based foragers respond more readily to heterospecific alarm calls than salliers, indicating greater reliance on social information (Goodale & Kotagama, 2008; Martínez, Gomez, Ponciano, & Robinson, 2016; Martínez & Zenil, 2012). Species with similar foraging behaviors convergently evolve similar morphological and physiological structures known as ecomorphs (Botero‐Delgadillo & Bayly, 2012; Corbin, 2008); for instance, eye morphology differs significantly between bird species in different foraging guilds (Lisney et al., 2013; Moore, Doppler, Young, & Fernández‐Juricic, 2013). These suites of physiological adaptations to foraging behaviors may result in similar physiological limitations on detection capability, and hence similar degrees of reliance on social information. We therefore predict that species which forage in more open microhabitats and employ more aerial foraging maneuvers will be less reliant on social information.

Alternatively, intraspecific sociality may play a key role in determining reliance on social information. Highly gregarious species living in groups may obtain most of their social information from group members, while solitary species must depend on heterospecifics. Social species are more likely to give alarm calls than solitary ones in order to warn group members or kin about predation risk (Sridhar, Beauchamp, & Shanker, 2009), whereas some solitary lizards, for example, lack alarm calls entirely (Fuong, Keeley, Bulut, & Blumstein, 2014). Social species also employ complex vigilance behaviors such as the sentinel systems which can accurately assess ambient predation risk through alarm calls (Ridley & Raihani, 2007; Ridley, Raihani, & Bell, 2010). Both birds and social primates only make use of heterospecific social information when in small conspecific groups, switching to conspecific social information in larger groups (Bshary & Noë, 1997; Ridley & Raihani, 2007). Solitary species, by contrast, often respond to heterospecific alarm calls of social species (Lea, Barrera, Tom, & Blumstein, 2008; Ridley, Wiley, & Thompson, 2014). We would predict, therefore, that intraspecifically social species will rely less on social information than solitary species.

Response to heterospecific alarm calls is also influenced by call relevance, or the proportion of instances in which the predator eliciting the alarm call represents a physical threat to the eavesdropper (Hua et al., 2016; Magrath et al., 2015). For example, arboreal hornbills are vulnerable to eagles but not leopards and only respond to the “eagle” alarm calls of a sympatric monkey species (Rainey, Zuberbuhler, & Slater, 2004a, 2004b). Similarly, New Holland honeyeaters (*Phylidonyris novaehollandiae*) respond to the alarm calls of white‐browed scrub‐wren (*Sericornis frontalis*; 18% of alarms given to nonshared predators), but not to those of superb fairy‐wrens (*Malurus cyaneus*; 52% of alarms given to nonshared predators, Magrath et al., 2009). Because the success and likelihood of attacks by predators are strongly influenced by predator–prey body‐size ratios, prey of similar body sizes will be vulnerable to the same predators (Rodgers, Downing, & Morrell, 2015). Therefore, the alarm‐caller‐to‐eavesdropper body‐size difference can serve as a proxy for the relevance of the call to the eavesdropper, and we would predict that species which are more similar in body size to the alarm caller will be more responsive to its alarm call.

The local context of an individual when an alarm is heard can also influence response to an alarm call because different foraging microhabitats have different associated predation risks (Brown & Kotler, 2004). The predators that prey on small forest passerines attack from above and target prey further from the trunk (Kullberg, 1995), so we predict that microsites will interact with species traits to define prey responses to simulated alarm calls. Density of vegetation and distance from trunk (Brotons, Orell, Lahti, & Koivula, 2000; Desrochers, 1989; Suhonen, 1993), height from ground (Carrascal & Alonso, 2006; Lee, Kuo, & Bollinger, 2005; Suhonen, 1993), and proximity to escape cover (Carrascal & Alonso, 2006; Lee et al., 2005) may all affect the perceived predation risk of forest passerines, and therefore, alarm call response might be greatest farther from escape cover and the trunk and closer to the ground. Perceived predation risk by small forest birds may also be higher in edge habitat than forest interior (Rodríguez, Andrén, & Jansson, 2001). Therefore, controlling for local microsite effects is important when attempting to make inferences from species‐level traits.

While the above factors have all been shown to influence response to alarm calls in isolation, their relative importance has never been simultaneously assessed in one community. In this study, we therefore present a common heterospecific alarm call from a sentinel species to a winter community of forest birds to elucidate the drivers of reliance on social information in a vertebrate eavesdropping network. We conduct a comparative test of the role of three species‐level ecological hypotheses (foraging ecology, sociality, and call relevance) in determining the degree of reliance on eavesdropping, while also controlling for local microhabitat effects.

## 2 METHODS

### 2.1 Study system

All field work was conducted on wildlands near Gainesville, Florida, USA, in the North‐central portion of the Florida peninsula. Study sites included San Felasco Hammock Preserve State Park (29°43′44″N 82°26′31″W), Paynes Prairie State Park (29°34′59″N 82°19′59″W), O'Leno State Park (29°55′01″N 82°35′02″W), Gum Root Park (29°40′50″N 82°14′17″W), and Newnan's Lake Conservation Area (29°40′58″N 82°13′29″W). We selected field sites only in upland hardwood forest, which has the most species‐rich winter forest bird community in Florida (Engstrom, 1993). These mesic, upland hardwood forests grow near lakes and spring‐fed steams and are dominated by an assemblage of deciduous trees (FNAI, 2010). Common canopy trees included American sweetgum (*Liquidambar styraciflua*), spruce pine (*Pinus glabra*), southern magnolia (*Magnolia grandiflora*), Florida maple (*Acer floridianum*), swamp chestnut oak (*Quercus michauxii*), diamondleaf oak (*Quercus laurifolia*), sugarberry (*Celtis laevigata*), and pignut hickory (*Carya glabra*).

Florida hosts a diverse winter bird community due to the presence of numerous short‐ and long‐distance migrants as well as resident species which vary widely in terms of foraging ecology, winter sociality, and body size (Kale & Maehr, 1990). It therefore offers an opportunity to test the influences of species traits over an extreme range of their values, yielding strong causal inference for each of our hypotheses (James & McCulloch, 1995). In this system, the information‐producing species is the Tufted Titmouse (*Baeolophus bicolor*, hereafter titmouse), an abundant, year‐round‐resident. It acts as a sentinel species through high vigilance combined with aggressive predator mobbing (Sieving, Contreras, & Maute, 2004) and alarm calling (Gaddis, 1980; Morse, 1970). Titmice produce complex alarm calls that accurately and reliably encode the size and threat level of a predator (Sieving, Hetrick, & Avery, 2010; Templeton et al., 2005) and thus have a community‐wide audience of eavesdroppers (Langham, Contreras, & Sieving, 2006; Sieving et al., 2004). Titmice also act as nuclear species for mixed‐species foraging flocks of birds (Contreras & Sieving, 2011) which form around small family groups that hold stable winter territories (Brawn & Samson, 1983). These foraging flocks are joined by many species of small forest passerines in winter that follow titmouse groups and forage with them (Farley, Sieving, & Contreras, 2008; Gaddis, 1983).

### 2.2 Characterizing foraging behavior

We obtained local data on the foraging ecology of the full winter community through focal individual field observations conducted during both winters at the same sites at which we conducted alarm call playbacks. Full methods are given in Jones, Sieving, and Robinson (2018); briefly, a single observer (HHJ) walked transects and trails at each site and recorded sequences of foraging behavior for each species encountered. We recorded sequences of foraging maneuvers until the focal individual was lost from view. For each foraging maneuver, we recorded the foraging height (estimated in meters using a laser rangefinder), the foraging maneuver type, the foraging substrate, the distance category from the trunk (near, medium, or far), and the foliage density at the microsite where the prey item was attacked (measured on a 0–5 scale). Foraging data were recorded in the field using a voice recorder, and later transcribed into a spreadsheet for analysis. Substrate and attack maneuver nomenclature follows Robinson and Remsen (1990), see Tables S5–S8 in Appendix S1 for proportional use of attack maneuver and microhabitat categories for each species. We performed foraging observations along a transect or trail only once per winter to avoid repeat foraging observations of the same individuals. For the same reason, when we encountered a mixed‐species foraging flock, we only recorded foraging observations of a single individual of each species in the flock.

### 2.3 Describing winter sociality and call relevance

Data on winter sociality and body mass were obtained from the literature. For the sociality data, we used the average maximum number of individuals of a species encountered in paired mixed‐species flock and point count surveys conducted at our field sites by Farley et al. (2008). Measuring winter sociality is challenging at our sites because species participate in mixed‐species foraging flocks, join single‐species foraging groups, or are solitary (Farley et al., 2008; Jones et al., 2018; Jones, Walters, & Robinson, 2019). Ten of 17 commonly occurring species at our study sites spend upwards of 80% of their time in mixed‐species flocks (Jones et al., 2019), so it is important to account for these foraging associations when quantifying winter sociality. Farley et al. (2008) therefore performed full surveys of flock composition paired with 10‐min point counts (without flocks present) in the path of the flock. They then averaged the maximum abundance of a given species detected during a flock survey with the maximum abundance of a species detected during a point count, as a proxy for the degree of heterospecific exclusion during the winter. Because species are not counter‐singing during the nonbreeding season in our system, detection of multiple individuals represents a combination of individuals joining mixed‐species flocks as a group, or multiple members of the same species associating socially outside of foraging flocks. Sociality of species in mixed‐species flocks is remarkably consistent within species in this system (Jones et al., 2019).

We used the absolute value of the difference in mass between the titmouse and each focal species as a proxy for the degree of overlap in predator suite, as the bird‐eating hawk species in our system (*Accipiter* spp.) preferentially prey on statistically different size classes of birds (Opdam, 1975; Reynolds & Meslow, 1984). Because body mass in birds affects vertical escape flight performance and other aspects of foraging and social behavior (Dial, Greene, & Irschick, 2008), difference in body mass should serve as a proxy for the difference in predation risk posed by a shared predator. Empirical data on *Accipiter* prey preferences from Europe support this assumption by suggesting that they prey less on species with very small and very large body masses in forest communities (Götmark & Post, 1996). Given the lack of published empirical data on relative prey preferences for North American *Accipiter* species on our focal community, we believe our measure represents our best estimate of the number of shared predators, with the relevance of the alarm call decreasing as the difference in mass increases. We obtained body mass estimates in grams from Sibley (2014).

### 2.4 Alarm call playback procedures

We conducted playback presentations from December 2014 to February 2015 (winter 1) and from November 2015 to January 2016 (winter 2) at the same field sites as foraging observations, though on different days. We used response to presentation of the titmouse Z call stimulus, an alarm call given by titmice in the presence of attacking hawks (Gaddis, 1980; Morse, 1970; Sieving et al., 2010), as a measure of reliance on social information. The call was presented in the absence of a predator, so species with complete personal information could “know” there was no predator, but species with limited personal information would be expected to respond. We did not present the alarm call with a predator model (e.g., taxidermied mount) because our methodology relied on presenting a “false” alarm call to the focal individual. Responding to false alarms is costly in terms of lost foraging efficiency (Bradbury & Vehrencamp, 2011), so we would expect for species to not respond when their personal information indicates that there is no predator. We selected our stimulus because it is a high‐urgency call, associated with the highest responsiveness by eavesdroppers (Fallow & Magrath, 2010). Even migratory species should be familiar with this stimulus because (a) most breeding ranges overlap with that of the titmouse (Sibley, 2014) and (b) birds can quickly learn novel alarm calls through acoustic association (Potvin, Ratnayake, Radford, & Magrath, 2018).

We presented free‐living, wild individuals of each bird species with the Z call, walking trails or transects until encountering a focal individual. We went into the field each day with a prioritized list of species needing more sampling, but otherwise, our sampling was opportunistic; we presented ten or more playbacks to each commonly encountered species in our nonbreeding community (sample sizes in Table 2). We only observed the response of a single focal bird, and in all cases, the observer remained at least 30 m from the focal bird to not influence behavior. The primary observer followed the focal bird with binoculars while a second observer set up and played the recording. Our recordings used known‐context alarm calls recorded during predator presentations to titmice in aviaries (Hetrick & Sieving, 2012). We created 30‐s playback recordings from natural calls (*N* = 5 exemplars) by repeating each natural recording with silence in between. We selected a random exemplar for each trial and played it at a standardized volume (~76 dBA at 1 m) from a speaker (Ultimate Ears BOOM) attached to an extension pole leaned on a tree (3.6 m height). The amplitude of the experimental exemplars was measured post hoc at 1 m in a similar habitat and using the same speaker and speaker settings (Table S12 in Appendix S1). Amplitude was measured as the maximum amplitude during the 30‐s recording in A‐weighted decibels using a digital sound level meter (B&K Precision 732A) on a fast setting. While the amplitude of natural Z calls is unknown, free‐living birds have been shown to respond to the same stimulus in the same habitat when given at ~50 dBA at 1 m (Grade & Sieving, 2016), so we feel confident that birds could hear our stimulus. We recorded the exemplar during the second winter only and therefore could not include it in our statistical analyses. However, this is a stereotyped alarm call only used in high‐risk contexts (Sieving et al., 2010), and we found no difference in response rates between exemplars (see Section 3; Tables S10–S11 in Appendix S1). Alarm call attenuation over greater signaler–receiver distances affects heterospecific response (Murray & Magrath, 2015), so we only used focal individuals within 30 m of the speaker (mean ± *SD*; 16.97 ± 6.01 m), as recorded by laser rangefinder prior to playback.

To maintain sample independence and minimize pseudo‐replication, we recorded a GPS point for each playback and separated all playbacks for each species, whether they were conducted on the same or different days, by at least 200 m. Playbacks conducted 200 m apart were also acoustically independent because the signal‐to‐noise ratio of Z calls degrades to 0 within 60 to 70 m of sound source in hardwood forests of the study region (K. E. Sieving, unpublished data). For each playback, we recorded three response variables: yes/no overall response, type of response (freezing in place or diving for cover), and length of freezing time if the bird froze. We scored a focal individual as responding if they immediately ceased baseline activity and adopted antipredatory behavior, while a response was scored as a no if the specific antipredatory behavior was not observed. Individuals respond to Z calls by either diving for cover or freezing in place and remaining motionless for two or more minutes (Gaddis, 1980; Hetrick & Sieving, 2012; Morse, 1970). Because response was immediate and behavioral changes were obvious and extended, it could never be confused with baseline behavior. Even stationary sit‐and‐wait flycatchers make noticeable and frequent head and body movements while scanning for prey while remaining on the same perch for extended periods of time. Therefore, we are confident that freezing behavior was not confused with even the most lethargic baseline behaviors exhibited by sit‐and‐wait flycatchers.

To describe the microsite of the focal individual at the time of playback, we recorded the density of vegetation (measured as the proportion of vegetation within a 1‐m radius sphere around the focal individuals; see Robinson & Remsen, 1990), distance from trunk (near, medium, or far), height from ground, and distance to escape cover (both estimated in meters using a rangefinder) before each playback. We classified playback locations based on whether they were located within 50 m of a forest edge (e.g., clear cut, pond edge) or not. Finally, we determined approximate temperature at time of playback post hoc using hourly averages at 10 m elevation for Gainesville from the Florida Automated Weather Network; temperature can influence vigilance levels in Holarctic parid‐led flocks (Brotons et al., 2000). To determine whether merely the presentation of any sound at the height of our speaker would startle birds into antipredator behavior, we performed a procedural control during the second winter using playback of the call of the spring peeper (*Pseudacris crucifer*). Procedural playbacks followed the same protocol and projected a call that sounded natural to us within 30 m (dBA at 1 m = ~80; Table S12 in Appendix S1). This small frog is a common resident of hardwood habitat and gives a somewhat similar high pitched, repeated call during its breeding season from November to March (Conant & Collins, 1998). As such, this represents a familiar, nonthreatening stimulus with similar acoustic qualities to the Z call.

### 2.5 Data reduction of foraging and microhabitat variables

All statistical analyses were performed in R (version 3.5.1). To describe covariance patterns and as a variable reduction technique, we performed Principal Coordinate Analysis (PCoA; Gower, 2015) on the foraging behavior data at the species level and the microhabitat data collected during playback at the individual level. Methods and interpretation of PCoA axes are described in detail in Appendix S2. Briefly, we ordinated the foraging and microhabitat data using the Gower dissimilarity index (Gower, 1971) to create a dissimilarity matrix. We selected axes to retain for further analyses (see Results in Appendix S2) by consulting a scree plot and retaining only interpretable axes.

### 2.6 Hypothesis evaluation using generalized linear models

We ran three generalized linear model (GLM; *glm* function, *stats* package) analyses of response to alarm call playback. First, we modeled (1) the overall (Y/N) response and (2) the response type‐dependent variables, using logit and log link GLMs, respectively. We did not model length of freezing response because we believe that resumption of baseline behavior is based on an “all clear” stimulus from the titmouse rather than species‐specific traits. Models of response type used the subset of the data in which the focal bird responded to the playback (*N* = 182 trials). We included eleven predictor variables (Table A1 in Appendix 1), encompassing both species‐level traits obtained from foraging observations and in the literature as well as local microhabitat data recorded before each playback. We included only complete cases in our analyses (*N* = 205 playback presentations).

In order to include a greater diversity of species in our analysis of playback response, we then ran (3) a second GLM model of response in which we included all species that received playbacks (*N* = 31 species, 238 playbacks), and a greater diversity of foraging behaviors and body masses. These additional species represent late migrants or rare overwintering species at our study sites, and since we did not have detailed foraging and sociality data for these taxa, we did not include them in the first analysis. We included seven predictor variables in the second model, including the local microhabitat variables, distance to speaker, difference in mass to the titmouse (e.g., call relevance), temperature, and foraging maneuver (see Table S3 in Appendix S1). Because foraging maneuver was the only important predictor variable for response in our first model, we classified it categorically by most common maneuver in the second model. Sociality was not included in the second model because it was not significant in the first model, and because we did not have local sociality data for the additional species. Foraging maneuver categories selected were either the most commonly observed maneuvers in foraging observations (Table S8 in Appendix S1) or the most common maneuver identified in the literature (see Table 1 for foraging maneuver categorization). We did not consider interactions in our models because main parameter estimates can be biased by interaction terms when model averaging (Richards, Whittingham, & Stephens, 2011).

**Table 1:** Summary of playback response

Species	*N*	Overall response	Freezing proportion	Mean freeze time (s)	Difference in mass	Mean local abundance	Foraging guild
ACFL	1	0.000			8.5		Sally
AMGO	5	1.000	1.000	106.20	8.5	3.17	Probe
AMRE	4	1.000	0.500	118.33	13.2		Sally‐hover
AMRO	1	1.000	1.000		55.5	2.87	Glean
BAWW	16	1.000	0.750	124.31	10.8	1.20	Glean
BGGN	14	0.786	0.364	203.56	15.5	3.89	Glean
BHVI	14	1.000	0.857	270.38	5.5	1.32	Glean
BLJA	4	0.750	0.667	129.33	63.5	1.82	Glean
BTBW	2	1.000	1.000	174.50	11.3		Sally‐hover
CACH	10	0.900	0.556	264.00	11	1.64	Glean
CAWR	8	0.875	0.571	236.00	0.5	1.43	Probe
CHSP	2	0.500	1.000		9.5	8.33	Reach
DOWO	9	1.000	1.000	183.78	5.5	1.41	Hammer
EAPH	16	0.063	0.000		1.5	1.18	Sally
EAWP	3	0.000			7.5		Sally
GRCA	2	1.000	0.000		15.5	1.33	Glean
HETH	10	1.000	1.000	451.20	9.5	1.20	Reach
MAWA	1	1.000	1.000		12.8		Glean
MYWA	13	1.000	1.000	116.91	9.2	4.57	Glean
NOCA	18	1.000	0.778	336.50	23.5	1.78	Reach
OCWA	11	1.000	0.818	259.30	12.5	1.00	Probe
OVEN	6	1.000	0.833	391.60	2	1.00	Reach
PIWA	9	1.000	0.778	170.71	9.5	3.84	Probe
PAWA	1	1.000	0.000		41.5		Glean
RBWO	11	0.727	0.875	250.43	15	1.51	Probe
RCKI	19	0.947	0.278	200.88	10	4.31	Glean
WEVI	10	1.000	0.700	163.43	25.5	1.24	Glean
WOTH	1	1.000	1.000		43.5		Probe
YBCU	1	0.000			28.5		Glean
YBSA	10	1.000	0.900	143.95	11.2	1.14	Hammer
YTWA	10	1.000	0.500	124.75	12.1	1.05	Probe

Note

Species codes are described in Table S9 in Appendix S1. Sample size = the number of Z call playbacks presented to each species. Overall Response = the proportion of individuals that responded by freezing or diving (vs. no change in behavior) to the playback stimulus. Freezing Proportion = the ratio of individuals for each species that froze versus dove (given a response). Mean Freeze Time = the mean number of seconds each species remained motionless (minimum of 2 responses to playback). Difference in Mass = absolute value of the difference in mass from the Tufted Titmouse (data from Sibley, 2014). Mean Local Abundance = a measure of the nonbreeding sociality of a species (calculated in Farley et al., 2008) Foraging Guild = foraging maneuver assigned to species based on field observations or data from the literature. The last three variables were used as predictor variables in the GLMs (see Table 2; Table S3 in Appendix S1).

We used an information theoretic approach (Burnham & Anderson, 2002) to evaluate our generalized linear models and determine the best models for each of our two response variables, using the Akaike information criterion modified for small sample sizes (AIC_c_), recommended for small datasets (Symonds & Moussalli, 2011). We calculated AIC_c_ scores and model weights for the full model set using the *dredge* function of the *MuMIn* package. Because there was no best model (model with a ΔAIC_c_ of 2 or greater over the second‐best model), we performed full model averaging over a candidate set of models (*model.avg* function, *MuMIn*). Because model weights were low, the 95% confidence set of models contained over 500 models. As such, we selected candidate sets with ΔAIC_c_ of 2 or less (Tables S1, S2, and S4 in Appendix S1) because these models are considered to be as good as the best model (Burnham & Anderson, 2002). The goodness of fit of models was assessed by the pseudo‐*r* ^2^ value calculated in the *dredge* function.

## 3 RESULTS

### 3.1 Foraging observations

Over two winters of observations, we observed 1,242 foraging maneuvers of 327 foraging individuals belonging to 25 species. Of these, 19 species had greater than 5 independent observations of foraging individuals (Table S8 in Appendix S1; full species‐level foraging data available in Tables S5–S7 in Appendix S1). The number of foraging observations was not biased by the average foraging height of a species (linear regression, *F* = 0.091, *df* = 13, *p = *.768) or the average vegetation density at which it forages (linear regression, *F* = 0.191, *df* = 13, *p = *.669). Similarly, the first foraging maneuver observed did not differ significantly from all foraging maneuvers (chi‐squared test, *χ* ^2^ = 18.49, *df* = 22, *p = *.679), which indicates that our foraging observations were not biased by more obvious foraging techniques. Generally, winter foraging behavior differed greatly between species (Table S8 in Appendix S1).

### 3.2 Playback experiment

We presented the alarm call stimulus to 242 individuals of 31 bird species, representing the entire winter bird community, and all species for which foraging data were collected (Table 1; full species names in Table S9 in Appendix S1). Of these, 16 species had nine or more playbacks, with the rest representing late southward migrants or rare species in the hardwood habitat. Individuals responded to 87% of presentations (*N* = 211), and 20 out of 31 species sampled (65%) responded to all stimuli. The exception was the Eastern Phoebe (*Sayornis phoebe*), a sit‐and‐wait flycatcher that rarely responded (6% response rate, *N* = 1 response) and represented 48% of the nonresponses to playback. A small subset of species responded primarily by diving, but the freezing response represented 152 of 211 playback responses (72%; Table 1). Length of freezing response, by contrast, showed strong intraspecific variation and weak interspecific variation, with averages ranging from 100 to 300 s (Table 1). For our procedural control, we performed 20 frog call playbacks to 11 species over the second winter, with no responses. We recorded the playback exemplar for 125 of 242 total trials (52%), all in the second winter. While the five exemplars were used at different frequencies (ANOVA, *F* = 2.67, *df* = 4, *p = *.04; Table S10 in Appendix S1), we found no statistical difference in response rates between exemplars when broken down by species (ANOVA, *F* = 0.72, *df* = 4, *p = *.58; Table S11 in Appendix S1).

### 3.3 Modeling determinants of reliance on social information

**Figure 1:**
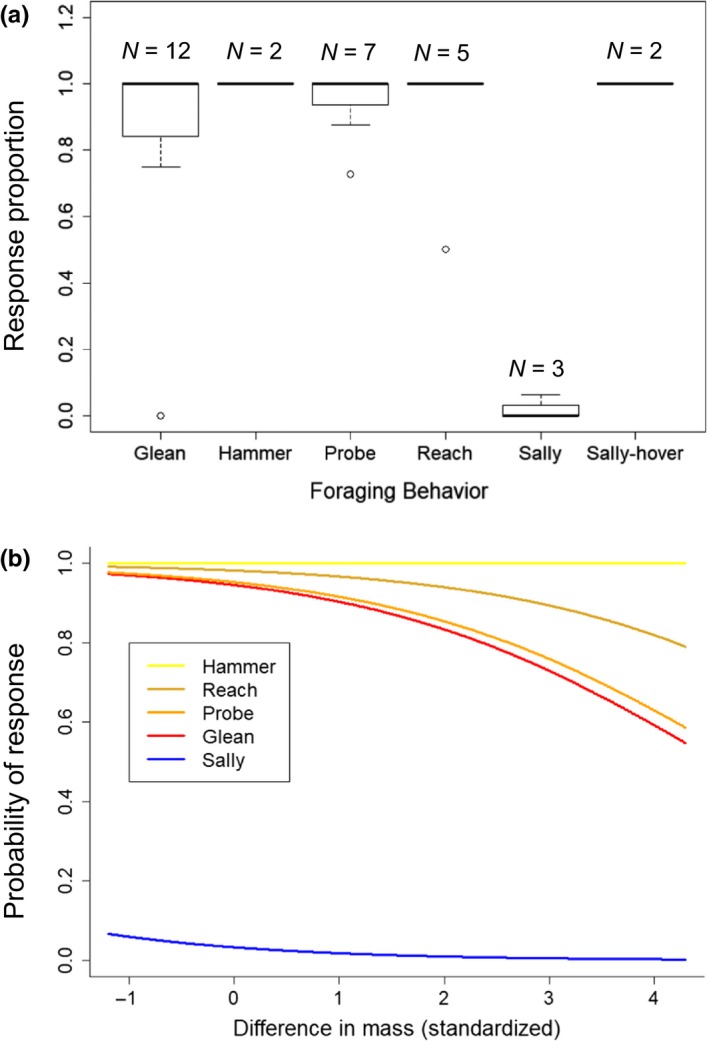
Importance of aerial foraging in determining playback response. Model results of overall response obtained from all species to which playback was presented (*N* = 31 species, 238 playbacks) are obtained from full model averaging of a candidate set of 8 generalized linear models (Table S4 in Appendix S1). (a) Species that forage more frequently using aerial sallying maneuvers responded to playback less often. Bolded line shows median response rate of all species in a foraging guild, and sample size indicates number of species. (b) Overall response rate to Z call playback is significantly lower for the sallying foraging maneuver (beta = −6.22, *p* = <.001) and at greater difference in mass from the titmouse (beta = −0.62, *p = *.003). Foraging maneuvers were assigned to species based on the most frequently observed foraging maneuver in foraging observations (Table S8 in Appendix S1) or based on values from the literature (Table 1). We did not include sociality in this model because it was not shown to be significant in the first model of overall response (Table 2). Full model results are shown in Table S3 in Appendix S1

Our model averaging results for overall playback response provide strong support for the foraging ecology hypothesis, as model averaging yielded a single significant foraging ecology predictor, the degree of aerial foraging (Aerial‐F, *p* = <.001, beta = −33.73; Table 2). The height at which a species forages also had a high beta estimate and was near significant (Height‐F, *p = *.095, beta = 11.18). By contrast, we found poor support for the sociality hypothesis (Sociality, *p = *.29, beta = 0.48) and our proxy for call relevance, difference in body mass, also had poor explanatory power (Difference in Mass, *p = *.13, beta = −0.07). When we modeled response using the expanded‐species pool, foraging ecology was similarly important, with sallying foragers showing a significant nonresponse when compared to gleaning species (Sally vs. Glean, *p* = <.001, beta = −6.22; Figure 1b and Table S3 in Appendix S1). However, difference in body mass also became a significant predictor once a larger sample size of body masses was included in the analysis (Difference in Mass, *p = *.003, beta = −0.62). Broadly, species that forage using aerial, sallying maneuvers are less likely to respond to the alarm call playback, and there was decreased responsiveness as body size increasingly differed from the titmouse. Our information theoretic approach yielded 20 candidate models for overall response with an average pseudo *r* ^2^ of 0.55 ± 0.01 (Table S1 in Appendix S1); the expanded‐species model set included 8 candidate models with an average pseudo *r* ^2^ of 0.62 ± 0.01 (Table S4 in Appendix S1).

**Table 2:** Model‐averaged results of GLMs of overall response and response type

Coefficient	Estimate	Standard error	Adjusted *SE*	*z* Value	*p*	Relative variable importance
Overall response (Y/N) *N* = 20 candidate models, Avg. pseudo‐*R* ^2^ = 0.55 ± 0.02						
Intercept	4.215	1.302	1.308	3.223	<.001	—
**Aerial‐F**	−33.725	8.014	8.061	4.184	<.001	1.00
Difference in mass	−0.074	0.049	0.049	1.498	.134	0.90
Edge‐MH	−2.928	2.241	2.249	1.302	.193	0.82
Escape‐MH	−3.255	3.442	3.453	0.943	.346	0.65
Height‐F	11.175	6.673	6.701	1.668	.095	0.96
Sociality	0.480	0.452	0.453	1.059	.290	0.67
Trunk‐F	8.357	7.853	7.874	1.061	.289	0.70
Distance to Speaker	−0.021	0.045	0.045	0.469	.639	0.28
Trunk‐MH	−0.360	1.299	1.303	0.277	.782	0.13
Occlusion‐F	1.058	3.759	3.772	0.280	.779	0.16
Response type (dive/freeze) *N* = 15 candidate models, Avg. pseudo‐*R* ^2^ = 0.21 ± 0.01						
Intercept	−0.110	0.689	0.693	0.159	.874	—
**Distance to Speaker**	0.069	0.033	0.033	2.076	.038	1.00
**Escape‐MH**	−5.025	1.920	1.933	2.600	.009	1.00
**Trunk‐F**	9.780	2.552	2.569	3.806	<.001	1.00
Temperature	0.011	0.023	0.023	0.484	.628	0.31
Height‐F	−0.846	1.924	1.931	0.438	.661	0.27
Edge‐MH	0.314	0.823	0.826	0.381	.704	0.24
Difference in mass	−0.006	0.016	0.016	0.370	.711	0.23
Sociality	0.008	0.052	0.052	0.159	.874	0.06
Aerial‐F	−0.373	1.827	1.834	0.203	.839	0.10
Trunk‐MH	−0.035	0.338	0.340	0.103	.918	0.05

Note

Bolded factors represent significant predictors, averaged over the candidate model set. Candidate models selected have a ΔAIC_c_ of 2 or less. The number of models in the candidate set for each response variable is indicated at the top of each table; for the response type analyses, we only included cases in which the individual responded to the stimulus. Reported pseudo‐*R* ^2^ values are the average ± *SD* of the McFadden's *R* ^2^ value for the candidate model set. Relative variable importance for each variable is calculated by summing the Akaike weights of the candidate models which include said variable. Predictor variable descriptions in Table A1 in Appendix 1.

**Figure 2:**
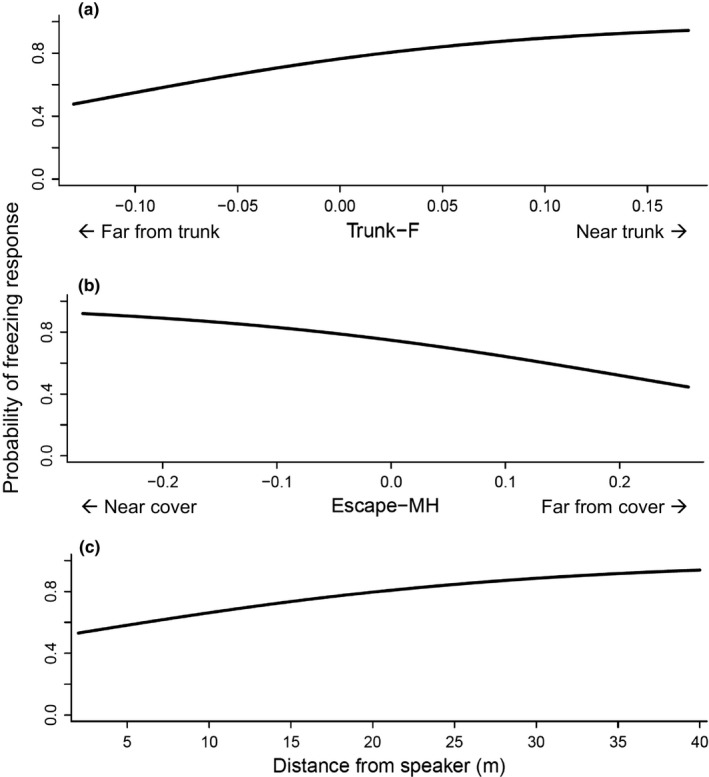
Fitted values for the significant predictors of response type (diving vs. freezing response). Significant predictors are obtained from model averaging of a candidate set of 15 generalized linear models with response type as the dependent variable (Table S2 in Appendix S1). We only analyzed cases in which the focal individual responded (*N* = 182 playbacks). Solid lines show probability of freezing response calculated by imputing values for the predictor variable of interest into the logistic regression equation for the full model (11 predictor variables; Table A1 in Appendix 1) and using the parameter estimate and intercept values from our model averaging (see Table 2). All other predictor variables were set to mean values. (a) Species which forage further from the trunk were more likely to dive than those that forage on or near the trunk. (b) Individuals foraging in more exposed microhabitats were more likely to dive than those closer to cover. (c) Individuals were more likely to dive for cover when located closer to the playback stimulus

For our models of response type, model averaging produced a single‐species‐level predictor of response type, the distance from the trunk at which a species forages (Trunk‐F; *p* = <.001, beta = 9.780; Table 2). However, local factors also affected response type, with the availability of escape cover for the focal individual (Escape‐MH; *p = *.01, beta = −5.03) and the distance of the focal individual from the playback speaker (Distance to Speaker; *p =* .04, beta = 0.07) also significant, though the effect size of distance to speaker was extremely small. Species that forage farther from the trunk were more likely to dive, whereas trunk‐foraging species were more likely to freeze (Figure 2a). Individuals located in exposed microhabitats that were farther from cover at the time of playback were more likely to dive than those located in sites with denser vegetation and closer to escape cover (Figure 2b). Finally, individuals were statistically more likely to freeze in place when they were located farther from the alarm call stimulus (Figure 2c). Our candidate set of models for response type consists of 15 models with an average pseudo‐*r* ^2^ of 0.21 ± 0.02 (Table S2 in Appendix S1).

## 4 DISCUSSION

### 4.1 Sit‐and‐wait salliers do not rely on social information

We determined that foraging ecology was the best of the three, species‐level hypotheses of the determinants of reliance on social information. The percentage of aerial foraging maneuvers (Aerial‐F) was the sole significant variable in explaining overall response in our first model (Table 2). While this trend was strongly driven by the Eastern Phoebe, we found a significant nonresponse by all sallying species when we expanded our model of playback response to include all species to which we performed playbacks (Figure 1a). The other two tyrannid flycatchers for which playback elicited no response (Acadian Flycatcher, *Empidonax virescens*; Eastern Wood Pewee, *Contopus virens*; Table 1) forage similarly using aerial sallies from a perch, while no other foraging maneuver type showed a significant effect on response (Table S3 in Appendix S1). While all nonresponding flycatchers are suboscines, we suggest that our findings highlight the uniqueness of aerial foraging behavior rather than an effect of phylogeny. Even in suboscine‐dominated Amazonian eavesdropping networks, substrate‐based foragers are more responsive to alarm calls than aerially foraging species, suggesting a unique nonresponse by aerial foragers (Martínez et al., 2016; Martínez & Zenil, 2012).

The nonresponse by sit‐and‐wait flycatchers may be explained by high visual acuity associated with the sit‐and‐wait sallying ecomorph. Inspection of wholemount retinas of sit‐and‐wait flycatchers (Tyrannidae) reveals high foveal neuron densities (Tyrrell & Fernández‐Juricic, 2017), as well as a cohort of giant retinal ganglion cells which are thought to be involved in movement detection (Coimbra, Luiza Videira Marceliano, Lara da Silveira Andrade‐da‐Costa, & Yamada, 2006). Such adaptations allow for high spatial resolution and visual acuity, enabling greater probability of predator detection and greater maximum and average detection distances (Tyrell & Fernández‐Juricic, 2015). Sallying species are known to act as sentinel species in both Neotropical (*Thamnomanes* antshrikes; Munn, 1986) and Paleotropical (*Dicrurus* drongos; Goodale & Kotagama, 2005a, 2005b) eavesdropping networks. Thus, eco‐morphological approaches, applied to visual ecology (e.g., Tyrell & Fernández‐Juricic, 2015), may be important in predicting the position of species in eavesdropping networks.

### 4.2 Species‐ and microhabitat‐specific antipredator behavior

Species‐level traits were more important than local microhabitat factors in determining reliance on social information. Local social and microhabitat factors were not significant predictors in either model for overall response, though the availability of escape cover (Escape‐MH) and distance from playback stimulus were significantly correlated with response type, suggesting that these factors influence *how* individuals respond. Small changes in microhabitat can greatly affect the cost of predation imposed on foraging prey species such as small forest passerines (Brown & Kotler, 2004), and shifts in microhabitat use have been documented under changing predation regimes in both time and space (Rodríguez et al., 2001; Suhonen, 1993). However, because foraging and vigilance behaviors vary from mutually exclusive in substrate‐based foragers to simultaneous activities in aerial foragers (Lima & Dill, 1990), the physiological trade‐offs between foraging mode and specialized eye morphology may be more important in driving reliance on social information (Guillemain, Martin, & Fritz, 2002).

The type of escape behavior adopted by responding individuals was species‐specific and dependent upon foraging microhabitat. Distance from trunk of the foraging microhabitat was the main driver: Trunk‐foraging species were more likely to freeze, while those foraging in the outer branches were more likely to dive. Outer‐branch‐foraging species need to dive for escape refugia to escape an attack (Lima, 1993), as microhabitats farther from the trunk are more exposed to small raptors (Kullberg, 1995). By contrast, trunk‐foraging species are less exposed to direct attack and can freeze to avoid detection. The feeding substrate also acts as a refuge by shielding the individual from an attacking predator (Lima, 1992): Woodpeckers typically freeze against the trunk and place themselves on the opposite side of the trunk from the playback speaker (Sullivan, 1984; H. H. Jones, personal observation). This mirrors the previously reported escape behaviors of foliage‐gleaning and bark‐foraging species (Lima, 1993) and provides more evidence that antipredator behavior is highly species‐specific and adapted to foraging microhabitat (Lima, 1992; Vanhooydonck & Van Damme, 2003). In birds, escape behavior may be taxonomically stereotyped—at least some species appear to lack the plasticity to adjust it in novel microhabitats (Koivula & Rönkä, 1998).

### 4.3 “Call relevance” influences response, sociality does not

We found empirical support for the call relevance hypothesis within our system, as measured by difference in body size between each focal species and the titmouse, when we modeled response across a larger range of difference in body masses (0.5–55.5 g, Table 1). This result mirrors trends observed in Australian and Sumatran eavesdropping networks, where similar‐sized species were also more responsive to the sentinel species’ alarm call (Hua et al., 2016; Magrath et al., 2009). We only detected this effect when including larger‐bodied species in our analysis, though the importance of call relevance in our system might be mediated by the information content of alarm calls, which can differ significantly between nuclear species (Goodale & Kotagama, 2005a). The greater information content of parid alarm calls, which encode more information about the size and threat level of a predator than those of heterospecifics (Sieving et al., 2010; Templeton et al., 2005), may increase the eavesdropping audience because this information can be relevant even to species that share fewer predators. Alternatively, our proxy measure of call relevance may be failing to account for true overlap in the predation risk. *Accipiter* hawks in Europe, for example, select prey in greater proportion to abundance based on habitat preferences and migratory habit (Götmark & Post, 1996), which we do not measure.

Sociality was not an important factor in determining degree of reliance on social information in our Florida winter community, in contrast to findings from tropical Africa (Radford & Ridley, 2007; Ridley & Raihani, 2007; Ridley et al., 2014). However, these findings come from social systems that comprise kin groups, such as many African primates and the Pied Babbler (*Turdoides bicolor*). Delayed dispersal and cooperative breeding is more common in the tropics (Brown, 1987), and the resulting family groups often become leaders of mixed‐species foraging flocks—possibly because of their alarm call systems (Sridhar & Shanker, 2014). By contrast, the species that form single‐species flocks in Florida (e.g., American Goldfinch, *Spinus tristis*; Yellow‐rumped Warbler, *Setophaga coronata*) are short‐distance migrants that form seasonal and temporary groups of nonkin individuals in winter (Hunt & Flaspohler, 1998; Prescott & Middleton, 1990). Thus, these species likely lack sentinel individuals and complex alarm call systems, reducing access to conspecific social information.

### 4.4 Parids as community informants

In sum, we found a strikingly near‐universal response to the Z call, highlighting the important role of the titmouse as an antipredator sentinel. Responsive species exhibit various migratory strategies and flocking propensities, and, in all, 28 species responded of the 31 tested. To our knowledge, this is the first empirical documentation of community‐wide responsiveness to the alarm call of a single sentinel species. Our results agree with previous findings of community‐wide responsiveness to the antipredator mobbing calls of species of the family Paridae (Gunn, Desrochers, Villard, Bourque, & Ibarzabal, 2000; Hurd, 1996; Langham et al., 2006; Sieving et al., 2004). Taken together, this reliance of the avian community on the social information of one species suggests a keystone role for the titmouse (sensu Kotliar, Baker, Whicker, & Plumb, 1999), likely because it provides highly reliable and complex information about predation risk (Hetrick & Sieving, 2012; Sieving et al., 2010; Templeton et al., 2005). Our results therefore support the idea that titmice play a keystone “community informant” role (Szymkowiack, 2013).

## CONFLICT OF INTEREST

Both authors gave final approval for publication and have no competing interests.

## AUTHORS' CONTRIBUTIONS

HHJ conceived the study, helped design the study, carried out the field work and statistical analyses, and drafted the manuscript. KES helped with the study design, statistical analyses, and manuscript drafting.

## Data Availability

The original datasets of Z call playbacks and winter foraging observations are archived in the University of Florida Digital Collections (accession numbers IR00010287 and IR00010286). Four electronic supplemental materials are made available: (a) Appendix S1 which consists of supplemental tables and figures, (b) Appendix S2 which includes methods, results, and interpretation of the PCoA, (c) our original R code for statistical analyses and data manipulation, and (d) the final dataset used for the GLM analyses.

## Appendix 1

**Table A1:** Predictor variables used in the GLMs for overall (Y/N) response and response type exhibited in response to titmouse Z call stimulus

Hypothesis	Variable name	Interpretation	Source
Foraging Ecology	Trunk‐F	Distance of species' foraging niche from trunk	PCoA: Foraging data
Foraging Ecology	Occlusion‐F	Visual occlusion of species' foraging niche	PCoA: Foraging data
Foraging Ecology	Height‐F	Foraging height above ground for each species' foraging niche	PCoA: Foraging data
Foraging Ecology	Aerial‐F	Degree of aerial foraging of each species' foraging niche	PCoA: Foraging data
Local Microhabitat	Edge‐MH	Forest edge versus interior at playback site	PCoA: Microhabitat recorded at playback
Local Microhabitat	Trunk‐MH	Distance of individual from trunk during playback	PCoA: Microhabitat recorded at playback
Local Microhabitat	Escape‐MH	Availability of escape cover during playback	PCoA: Microhabitat recorded at playback
Playback Procedure	Distance to Speaker	Distance in meters from speaker to focal individual	Recorded prior to playback
Sociality	Sociality	Maximum abundance of each species in and out of mixed‐species foraging flocks	Farley et al. (2008)
Call Relevance	Difference in Mass	Absolute value of the difference in mass to the Tufted Titmouse	Calculated from Sibley (2014)
—	Temperature	Hourly temperature average at 10 m	Florida Automated Weather Network

Note

The first four variables represent principal coordinate axes obtained from ordination of field observations of foraging behavior (37 variables; Table S3 in Appendix S2) while the next three variables similarly represent principal coordinate axes obtained from an ordination of six microhabitat variables associated with the target individual recorded just before each playback (Table S4 in Appendix S2).

Abbreviations: F, foraging; MH, microhabitat.
