# Supplementary material for: Foraging ecology drives social information reliance in an avian eavesdropping community
Source: Ecol Evol. 2019 Sep 14;9(20):11584–97. doi: 10.1002/ece3.5561 (PMC6822049; doi:10.1002/ece3.5561)
Supplement: Supplementary file 1 [file ECE3-9-11584-s001.docx]

**Appendix S1: Supplemental Tables and Figures**

**Table S1. Candidate model set of 20 GLMs for overall response used in the model averaging.** Candidate models selected were those with a ΔAIC_c_ <2. Descriptions of model terms given in Table 10, Supplement S1.

| **Model Rank** | **Model Terms** | **McFadden's R^2^** | **Degrees of freedom** | **Log likelihood** | **AIC_c_** | **ΔAIC_c_** | **Akaike weight** |
| --- | --- | --- | --- | --- | --- | --- | --- |
| 1 | Aerial-F + Difference in Mass + Edge-MH + Escape-MH + Height-F + Sociality + Trunk-F | 0.569 | 8 | -37.30 | 91.33 | 0.00 | 0.017 |
| 2 | Aerial-F + Difference in Mass + Distance to Speaker + Edge-MH + Height-F + Sociality +Trunk-F | 0.564 | 8 | -37.62 | 91.97 | 0.64 | 0.012 |
| 3 | Aerial-F + Difference in Mass + Escape-MH + Height-F + Sociality + Trunk-F | 0.549 | 7 | -38.74 | 92.04 | 0.71 | 0.012 |
| 4 | Aerial-F + Difference in Mass + Edge-MH + Escape-MH + Height-F | 0.533 | 6 | -39.85 | 92.12 | 0.78 | 0.012 |
| 5 | Aerial-F + Difference in Mass + Edge-MH + Height-F | 0.517 | 5 | -40.97 | 92.24 | 0.91 | 0.011 |
| 6 | Aerial-F + Difference in Mass + Distance to Speaker + Edge-MH + Escape-MH + Height-F + Sociality + Trunk-F | 0.577 | 9 | -36.71 | 92.35 | 1.02 | 0.010 |
| 7 | Aerial-F + Difference in Mass + Edge-MH + Height-F + Sociality + Trunk-F | 0.547 | 7 | -38.91 | 92.38 | 1.05 | 0.010 |
| 8 | Aerial-F + Difference in Mass + Edge-MH + Escape-MH + Height-F + Sociality + Trunk-F + Trunk-MH | 0.577 | 9 | -36.74 | 92.39 | 1.06 | 0.010 |
| 9 | Aerial-F + Edge-MH + Escape-MH + Height-F | 0.516 | 5 | -41.08 | 92.47 | 1.14 | 0.010 |
| 10 | Aerial-F + Difference in Mass + Edge-MH + Escape-MH + Height-F + Occlusion-F + Sociality + Trunk-F | 0.575 | 9 | -36.88 | 92.68 | 1.35 | 0.009 |
| 11 | Aerial-F + Difference in Mass + Distance to Speaker + Edge-MH + Height-F | 0.529 | 6 | -40.16 | 92.75 | 1.42 | 0.008 |
| 12 | Aerial-F + Distance to Speaker + Edge-MH + Height-F | 0.513 | 5 | -41.26 | 92.81 | 1.48 | 0.008 |
| 13 | Aerial-F + Difference in Mass + Escape-MH + Height-F + Sociality + Trunk-F + Trunk-MH | 0.558 | 8 | -38.07 | 92.87 | 1.54 | 0.008 |
|  |  |  |  |  |  |  |  |
| **Table S1.** Continued. | |  |  |  |  |  |  |
|  |  |  |  |  |  |  |  |
| 14 | Aerial-F + Difference in Mass + Edge-MH + Escape-MH + Sociality + Trunk-F | 0.542 | 7 | -39.24 | 93.05 | 1.72 | 0.007 |
| 15 | Aerial-F + Difference in Mass + Edge-MH + Height-F + Occlusion-F + Sociality + Trunk-F | 0.556 | 8 | -38.20 | 93.13 | 1.80 | 0.007 |
| 16 | Aerial-F + Difference in Mass + Escape-MH + Height-F + Occlusion-F + Sociality + Trunk-F | 0.556 | 8 | -38.24 | 93.21 | 1.88 | 0.007 |
| 17 | Aerial-F + Difference in Mass + Distance to Speaker + Edge-MH + Height-F + Occlusion-F + Sociality + Trunk-F | 0.571 | 9 | -37.15 | 93.23 | 1.90 | 0.007 |
| 18 | Aerial-F + Difference in Mass + Edge-MH + Escape-MH + Height-F + Trunk-MH | 0.541 | 7 | -39.33 | 93.23 | 1.90 | 0.007 |
| 19 | Aerial-F + Difference in Mass + Distance to Speaker + Escape-MH + Height-F + Sociality + Trunk-F | 0.555 | 8 | -38.29 | 93.32 | 1.98 | 0.006 |
| 20 | Aerial-F + Difference in Mass + Edge-MH + Escape-MH + Height-F + Trunk-F | 0.540 | 7 | -39.38 | 93.32 | 1.99 | 0.006 |

**Table S2. Candidate set of best models to explain response type (dive versus freeze).** Candidate models selected were those with a ΔAIC_c_ <2. Descriptions of model terms given in Table 10, Supplement S1.

| **Model Rank** | **Model Terms** | **McFadden's R^2^** | **Degrees of freedom** | **Log likelihood** | **AIC_c_** | **ΔAIC_c_** | **Akaike weight** |
| --- | --- | --- | --- | --- | --- | --- | --- |
| 1 | Distance to Speaker + Escape-MH + Trunk-F | 0.203 | 4 | -94.97 | 198.16 | 0.00 | 0.022 |
| 2 | Distance to Speaker + Escape-MH + Temperature + Trunk-F | 0.214 | 5 | -94.15 | 198.64 | 0.48 | 0.017 |
| 3 | Distance to Speaker + Escape-MH + Height-F + Trunk-F | 0.214 | 5 | -94.20 | 198.74 | 0.59 | 0.016 |
| 4 | Distance to Speaker + Edge-MH + Escape-MH + Trunk-F | 0.212 | 5 | -94.34 | 199.03 | 0.87 | 0.014 |
| 5 | Difference in Mass + Distance to Speaker + Escape-MH + Trunk-F | 0.210 | 5 | -94.45 | 199.25 | 1.09 | 0.013 |
| 6 | Distance to Speaker + Escape-MH + Height-F + Temperature + Trunk-F | 0.223 | 6 | -93.48 | 199.44 | 1.29 | 0.012 |
| 7 | Distance to Speaker + Escape-MH + Sociality + Trunk-F | 0.208 | 5 | -94.61 | 199.56 | 1.40 | 0.011 |
| 8 | Distance to Speaker + Edge-MH + Escape-MH + Height-F + Trunk-F | 0.222 | 6 | -93.57 | 199.63 | 1.47 | 0.011 |
| 9 | Difference in Mass + Distance to Speaker + Escape-MH + Height-F + Trunk-F | 0.222 | 6 | -93.59 | 199.66 | 1.50 | 0.010 |
| 10 | Aerial-F + Distance to Speaker + Escape-MH + Trunk-F | 0.207 | 5 | -94.67 | 199.68 | 1.52 | 0.010 |
| 11 | Difference in Mass + Distance to Speaker + Escape-MH + Temperature + Trunk-F | 0.221 | 6 | -93.61 | 199.70 | 1.54 | 0.010 |
| 12 | Distance to Speaker + Edge-MH + Escape-MH +Temperature + Trunk-F | 0.221 | 6 | -93.64 | 199.76 | 1.60 | 0.010 |
| 13 | Distance to Speaker + Escape-MH + Trunk-F + Trunk-MH | 0.205 | 5 | -94.82 | 199.99 | 1.83 | 0.009 |
| 14 | Aerial-F + Distance to Speaker + Escape-MH + Temperature + Trunk-F | 0.219 | 6 | -93.81 | 200.10 | 1.94 | 0.008 |
| 15 | Difference in Mass + Distance to Speaker + Edge-MH + Escape-MH + Trunk-F | 0.219 | 6 | -93.81 | 200.10 | 1.94 | 0.008 |

**Table S3. Model-averaged results of GLMs of overall response extended to all species.** Bolded factors represent significant predictors, averaged over the candidate model set. Candidate models selected have a ΔAIC_c_ of 2 or less. Reported pseudo-r^2^ values are the average ± SD of the McFadden’s R^2^ value for the candidate model set. Relative variable importance for each variable is calculated by summing the Akaike weights of the candidate models which include said variable. Predictor variable descriptions in Table 10, Supplement S1; foraging was assigned as a categorical variable to each species based on most frequent foraging maneuver (foraging maneuver categories for all species listed in Table 1). Results for the foraging maneuver are presented as statistical difference in response between the most common maneuver (Glean) and each other maneuver.

| **Overall Response (all species)** | | | | | | |
| --- | --- | --- | --- | --- | --- | --- |
| N = 8 candidate models, Avg. pseudo-r^2^ = 0.62 ± 0.01 | | | | | | |
| **Coefficient** | **Estimate** | **Standard Error** | **Adjusted SE** | **z value** | **p** | **Relative variable importance** |
| Intercept | 2.844 | 0.441 | 0.443 | 6.413 | <0.001 | - |
| **Difference in Mass** | -0.619 | 0.205 | 0.206 | 3.009 | 0.003 | 1.00 |
| Escape-MH | -4.378 | 2.923 | 2.939 | 1.490 | 0.136 | 0.59 |
| Edge-MH | -2.325 | 1.873 | 1.883 | 1.235 | 0.217 | 0.35 |
| Trunk-MH | -2.158 | 2.196 | 2.208 | 0.977 | 0.328 | 0.28 |
| Distance to Speaker | 0.187 | 0.349 | 0.351 | 0.531 | 0.595 | 0.08 |
| Foraging: |  |  |  |  |  | 1.00 |
| Glean vs. Hammer | 16.116 | 1442.103 | 1449.741 | 0.011 | 0.991 |  |
| Glean vs. Probe | 0.158 | 0.716 | 0.719 | 0.220 | 0.826 |  |
| Glean vs. Reach | 1.139 | 1.142 | 1.148 | 0.992 | 0.321 |  |
| **Glean vs. Sally** | -6.224 | 1.164 | 1.170 | 5.318 | <0.001 |  |
| Glean vs. Sally-hover | 15.749 | 2632.151 | 2646.092 | 0.006 | 0.995 |  |

**Table S4. Candidate set of 8 best models to explain overall response when expanded to all species.** Candidate models selected were those with a ΔAIC_c_ < 2. Descriptions of model terms given in Table 10, Supplement S1.

| **Model Rank** | **Model Terms** | **McFadden's R^2^** | **Degrees of freedom** | **Log likelihood** | **AIC_c_** | **ΔAIC_c_** | **Akaike weight** |
| --- | --- | --- | --- | --- | --- | --- | --- |
| 1 | Difference in Mass + Escape-MH + Foraging | 0.615 | 8 | -43.11 | 102.85 | 0.00 | 0.10 |
| 2 | Difference in Mass + Foraging | 0.602 | 7 | -44.27 | 103.02 | 0.17 | 0.09 |
| 3 | Difference in Mass + Escape-MH + Edge-MH + Foraging | 0.623 | 9 | -42.34 | 103.47 | 0.62 | 0.07 |
| 4 | Difference in Mass + Edge-MH + Foraging | 0.611 | 8 | -43.48 | 103.58 | 0.73 | 0.07 |
| 5 | Difference in Mass + Escape-MH + Foraging + Trunk-MH | 0.620 | 9 | -42.61 | 103.10 | 1.15 | 0.06 |
| 6 | Difference in Mass + Foraging + Trunk-MH | 0.607 | 8 | -43.80 | 104.23 | 1.38 | 0.05 |
| 7 | Difference in Mass + Edge-MH + Escape-MH + Foraging + Trunk-MH | 0.628 | 10 | -41.86 | 104.69 | 1.85 | 0.04 |
| 8 | Difference in Mass + Distance to Speaker + Escape-MH + Foraging | 0.616 | 9 | -42.96 | 104.72 | 1.87 | 0.04 |

**Table S5. Proportions of foraging substrate use in a winter bird community.** Foraging observations were made from December to February in hardwood forests near Gainesville, Florida, USA. Foraging substrates are categorized after Remsen and Robinson’s (1990) schema. Substrate, vegetation density, height from ground, and distance from trunk were recorded for each attack maneuver. Species names correspond to the four-letter Alpha Codes, as determined by the American Ornithologists’ Union (see Table 9, Supplement S1 for full species names).

| **Species** | **Foraging Substrates** | | | | | | | | | | | |
| --- | --- | --- | --- | --- | --- | --- | --- | --- | --- | --- | --- | --- |
|  | **Air** | **Branch** | **Dead Branch** | **Dead Leaf** | **Epiphyte** | **Fruiting Body** | **Ground** | **Live Leaf** | **Pine Cone** | **Pine Needles** | **Trunk** | **Vine** |
| AMGO | 0.0000 | 0.0000 | 0.0000 | 0.0000 | 0.0000 | 1.0000 | 0.0000 | 0.0000 | 0.0000 | 0.0000 | 0.0000 | 0.0000 |
| BAWW | 0.0184 | 0.0859 | 0.0552 | 0.0061 | 0.0736 | 0.0000 | 0.0000 | 0.0000 | 0.0000 | 0.0000 | 0.7362 | 0.0245 |
| BGGN | 0.1789 | 0.2316 | 0.0000 | 0.0105 | 0.0842 | 0.0000 | 0.0000 | 0.4842 | 0.0000 | 0.0000 | 0.0105 | 0.0000 |
| BHVI | 0.0245 | 0.4878 | 0.0244 | 0.0000 | 0.0488 | 0.0000 | 0.0000 | 0.0976 | 0.0000 | 0.0000 | 0.1951 | 0.1220 |
| CACH | 0.0000 | 0.7381 | 0.0714 | 0.0238 | 0.0000 | 0.0238 | 0.0000 | 0.1190 | 0.0000 | 0.0000 | 0.0238 | 0.0000 |
| CAWR | 0.0000 | 0.1111 | 0.0556 | 0.5556 | 0.0556 | 0.0000 | 0.0000 | 0.1111 | 0.0000 | 0.0000 | 0.0556 | 0.0556 |
| DOWO | 0.0000 | 0.2295 | 0.1803 | 0.0000 | 0.0656 | 0.0000 | 0.0000 | 0.0000 | 0.0000 | 0.0000 | 0.5246 | 0.0000 |
| EAPH | 0.2050 | 0.2941 | 0.0294 | 0.0882 | 0.0000 | 0.0000 | 0.0588 | 0.2059 | 0.0000 | 0.0294 | 0.0882 | 0.0000 |
| MYWA | 0.0986 | 0.2817 | 0.0000 | 0.0704 | 0.0282 | 0.0423 | 0.0000 | 0.2676 | 0.0000 | 0.0000 | 0.1268 | 0.0845 |
| NOCA | 0.0000 | 0.2340 | 0.0000 | 0.0000 | 0.0000 | 0.0000 | 0.6170 | 0.1489 | 0.0000 | 0.0000 | 0.0000 | 0.0000 |
| OCWA | 0.0000 | 0.0153 | 0.0000 | 0.7538 | 0.0462 | 0.0462 | 0.0000 | 0.1385 | 0.0000 | 0.0000 | 0.0000 | 0.0000 |
| OVEN | 0.0000 | 0.0000 | 0.0000 | 0.0000 | 0.0000 | 0.0000 | 0.9615 | 0.0385 | 0.0000 | 0.0000 | 0.0000 | 0.0000 |
| PIWA | 0.0278 | 0.1389 | 0.0000 | 0.1528 | 0.3472 | 0.0278 | 0.0278 | 0.0972 | 0.0139 | 0.0972 | 0.0694 | 0.0000 |
| RBWO | 0.0000 | 0.2400 | 0.2800 | 0.0000 | 0.0500 | 0.0000 | 0.0000 | 0.0100 | 0.0000 | 0.0000 | 0.3800 | 0.0000 |
| RCKI | 0.0272 | 0.1837 | 0.0000 | 0.0000 | 0.0000 | 0.0000 | 0.0000 | 0.7551 | 0.0000 | 0.0068 | 0.0204 | 0.0000 |
| TUTI | 0.0000 | 0.3393 | 0.1607 | 0.2321 | 0.1250 | 0.0000 | 0.0000 | 0.0893 | 0.0000 | 0.0000 | 0.0000 | 0.0536 |
| WEVI | 0.0333 | 0.1667 | 0.0000 | 0.0000 | 0.1000 | 0.0000 | 0.0000 | 0.6333 | 0.0000 | 0.0000 | 0.0000 | 0.0667 |
| YBSA | 0.0000 | 0.0000 | 0.0000 | 0.0000 | 0.0000 | 0.0000 | 0.0000 | 0.0000 | 0.0000 | 0.0000 | 0.9149 | 0.0851 |
| YTWA | 0.0145 | 0.0870 | 0.0290 | 0.3623 | 0.2174 | 0.0145 | 0.0000 | 0.0145 | 0.1159 | 0.0435 | 0.0580 | 0.0435 |

**Table S6.** **Proportions of use of distance from trunk and foliage density categories in a winter bird community.** Foraging observations were made from December to February in hardwood forests near Gainesville, Florida, USA. Substrate, vegetation density, height from ground, and distance from trunk were recorded for each attack maneuver. Following Robinson and Remsen (1990), we categorized vegetation density on a 0-5 scale based on the amount of visible light in a 1-meter-diameter sphere around the focal individual at the time of the attack maneuver. Distance from trunk was binned into three categories (near, medium, far) according to the same schema. Species names correspond to the four-letter Alpha Codes, as determined by the American Ornithologists’ Union (see Table 9, Supplement S1 for full species names).

| **Species** | **Distance from Trunk** | | | **Foliage Density** | | | | |
| --- | --- | --- | --- | --- | --- | --- | --- | --- |
|  | **Far** | **Medium** | **Near** | **0** | **1** | **2** | **3** | **4** |
| AMGO | 0.7692 | 0.0000 | 0.2308 | 0.0000 | 0.5385 | 0.4615 | 0.0000 | 0.0000 |
| BAWW | 0.0798 | 0.1963 | 0.7239 | 0.1595 | 0.4540 | 0.3190 | 0.0675 | 0.0000 |
| BGGN | 0.4608 | 0.3039 | 0.2353 | 0.0297 | 0.1980 | 0.2970 | 0.4158 | 0.0594 |
| BHVI | 0.2857 | 0.3571 | 0.3571 | 0.0238 | 0.5952 | 0.2381 | 0.1429 | 0.0000 |
| CACH | 0.5882 | 0.2353 | 0.1765 | 0.0256 | 0.1538 | 0.2308 | 0.5128 | 0.0769 |
| CAWR | 0.2000 | 0.0000 | 0.8000 | 0.0000 | 0.1111 | 0.3889 | 0.2778 | 0.2222 |
| DOWO | 0.2787 | 0.2295 | 0.4918 | 0.0492 | 0.7377 | 0.1967 | 0.0164 | 0.0000 |
| EAPH | 0.5600 | 0.2400 | 0.2000 | 0.1515 | 0.3333 | 0.3333 | 0.1818 | 0.0000 |
| MYWA | 0.2951 | 0.3443 | 0.3607 | 0.0571 | 0.2286 | 0.2429 | 0.3429 | 0.1286 |
| NOCA | 0.5455 | 0.4545 | 0.0000 | 0.2979 | 0.2340 | 0.1702 | 0.2340 | 0.0638 |
| OCWA | 0.4426 | 0.3115 | 0.2459 | 0.0000 | 0.1875 | 0.3281 | 0.4688 | 0.0156 |
| OVEN | NA | NA | NA | 0.0000 | 0.2692 | 0.3846 | 0.1538 | 0.1923 |
| PIWA | 0.4058 | 0.2464 | 0.3478 | 0.0286 | 0.2714 | 0.3714 | 0.3000 | 0.0286 |
| RBWO | 0.0900 | 0.3700 | 0.5400 | 0.0104 | 0.6771 | 0.1979 | 0.1146 | 0.0000 |
| RCKI | 0.5664 | 0.2308 | 0.2028 | 0.0200 | 0.0666 | 0.3733 | 0.4867 | 0.0533 |
| TUTI | 0.4423 | 0.3462 | 0.2115 | 0.0000 | 0.0600 | 0.38 | 0.4600 | 0.1000 |
| WEVI | 0.5000 | 0.2143 | 0.2857 | 0.0000 | 0.0670 | 0.2414 | 0.5862 | 0.1034 |
| YBSA | 0.0000 | 0.1702 | 0.8298 | 0.2128 | 0.5957 | 0.0638 | 0.0000 | 0.1277 |
| YTWA | 0.4265 | 0.3382 | 0.2353 | 0.0000 | 0.2647 | 0.2794 | 0.3529 | 0.1029 |

**Table S7.** **Proportions of foraging maneuver use and mean foraging heights of a winter bird community.** All foraging observations were made in Florida upland hardwood forest between December and February. Foraging attack maneuvers are classified according to the Robinson and Remsen (1990) typology. Mean foraging heights are measured as meters above the ground and are averaged across all foraging observations. Species names correspond to the American Ornithologists’ Union’s four-letter Alpha Codes (see Table 9, Supplement S1 for full species names).

| **Species** | **Foraging Maneuvers** | | | | | | | | | |
| --- | --- | --- | --- | --- | --- | --- | --- | --- | --- | --- |
|  | **Flake** | **Flush-pursue** | **Gape** | **Glean** | **Hammer** | **Hang** | **Hang-down probe** | **Lunge** | **Peck** | **Probe** |
| AMGO | 0.0000 | 0.0000 | 0.0000 | 0.0000 | 0.0000 | 0.0000 | 0.0769 | 0.0000 | 0.0000 | 0.9231 |
| BAWW | 0.0184 | 0.0184 | 0.0000 | 0.7914 | 0.0000 | 0.0122 | 0.0061 | 0.0000 | 0.0000 | 0.1411 |
| BGGN | 0.0000 | 0.1667 | 0.0000 | 0.5000 | 0.0000 | 0.0098 | 0.0000 | 0.0000 | 0.0000 | 0.0098 |
| BHVI | 0.0000 | 0.0238 | 0.0000 | 0.4048 | 0.0000 | 0.0238 | 0.0000 | 0.0000 | 0.0000 | 0.0238 |
| CACH | 0.0000 | 0.0000 | 0.0000 | 0.3333 | 0.1905 | 0.2856 | 0.0000 | 0.0000 | 0.0000 | 0.0476 |
| CAWR | 0.0000 | 0.0000 | 0.0000 | 0.3333 | 0.0000 | 0.0000 | 0.0000 | 0.0000 | 0.0000 | 0.5556 |
| DOWO | 0.0328 | 0.0000 | 0.0000 | 0.1311 | 0.6885 | 0.0164 | 0.0000 | 0.0000 | 0.0820 | 0.0492 |
| EAPH | 0.0000 | 0.0000 | 0.0000 | 0.0000 | 0.0000 | 0.0000 | 0.0000 | 0.0000 | 0.0000 | 0.0000 |
| MYWA | 0.0000 | 0.0282 | 0.0000 | 0.5634 | 0.0000 | 0.0000 | 0.0000 | 0.0000 | 0.0000 | 0.0845 |
| NOCA | 0.0000 | 0.0000 | 0.0000 | 0.4468 | 0.0000 | 0.0000 | 0.0000 | 0.0213 | 0.0000 | 0.0000 |
| OCWA | 0.0154 | 0.0000 | 0.0154 | 0.0769 | 0.0000 | 0.0154 | 0.0308 | 0.0000 | 0.0000 | 0.6769 |
| OVEN | 0.0000 | 0.0000 | 0.0000 | 0.0384 | 0.0000 | 0.0000 | 0.0000 | 0.0769 | 0.0000 | 0.0000 |
| PIWA | 0.0278 | 0.0278 | 0.0000 | 0.3750 | 0.0000 | 0.0278 | 0.0139 | 0.0000 | 0.0000 | 0.4028 |
| RBWO | 0.0400 | 0.0000 | 0.0000 | 0.0000 | 0.1700 | 0.0000 | 0.0000 | 0.0000 | 0.1100 | 0.6000 |
| RCKI | 0.0000 | 0.0250 | 0.0000 | 0.5063 | 0.0000 | 0.0189 | 0.0000 | 0.0000 | 0.0000 | 0.0000 |
| TUTI | 0.0714 | 0.0000 | 0.0000 | 0.2679 | 0.1071 | 0.1786 | 0.0000 | 0.0000 | 0.0000 | 0.2679 |
| WEVI | 0.0000 | 0.0333 | 0.0000 | 0.5333 | 0.0000 | 0.1000 | 0.0000 | 0.0000 | 0.0000 | 0.0000 |
| YBSA | 0.0000 | 0.0000 | 0.0000 | 0.0213 | 0.7660 | 0.0000 | 0.0000 | 0.0000 | 0.0000 | 0.2128 |
| YTWA | 0.0000 | 0.0145 | 0.0000 | 0.1449 | 0.0000 | 0.0290 | 0.0145 | 0.0000 | 0.0000 | 0.6812 |

**Table S7.** Continued.

| **Species** | **Pry** | **Pull** | **Reach** | **Reach-down probe** | **Sally** | **Sally-hover** | **Sally-pounce** | **Mean foraging height (m)** |
| --- | --- | --- | --- | --- | --- | --- | --- | --- |
| AMGO | 0.0000 | 0.0000 | 0.0000 | 0.0000 | 0.0000 | 0.0000 | 0.0000 | 21.3077 |
| BAWW | 0.0000 | 0.0000 | 0.0000 | 0.0000 | 0.0000 | 0.0061 | 0.0061 | 10.7761 |
| BGGN | 0.0000 | 0.0000 | 0.0882 | 0.0000 | 0.0196 | 0.1765 | 0.0294 | 12.5510 |
| BHVI | 0.0000 | 0.0000 | 0.0714 | 0.0000 | 0.0238 | 0.2857 | 0.1429 | 11.2262 |
| CACH | 0.0000 | 0.0000 | 0.0476 | 0.0000 | 0.0000 | 0.0000 | 0.0000 | 12.1750 |
| CAWR | 0.0000 | 0.0000 | 0.1111 | 0.0000 | 0.0000 | 0.0000 | 0.0000 | 4.1667 |
| DOWO | 0.0000 | 0.0000 | 0.0000 | 0.0000 | 0.0000 | 0.0000 | 0.0000 | 16.0481 |
| EAPH | 0.0000 | 0.0000 | 0.0882 | 0.0000 | 0.5882 | 0.2059 | 0.1176 | 10.3240 |
| MYWA | 0.0000 | 0.0000 | 0.1126 | 0.0000 | 0.1127 | 0.0845 | 0.0141 | 9.9203 |
| NOCA | 0.0000 | 0.0000 | 0.5319 | 0.0000 | 0.0000 | 0.0000 | 0.0000 | 4.2766 |
| OCWA | 0.0000 | 0.0000 | 0.0462 | 0.1231 | 0.0000 | 0.0000 | 0.0000 | 9.6563 |
| OVEN | 0.0000 | 0.0000 | 0.8846 | 0.0000 | 0.0000 | 0.0000 | 0.0000 | 0.0000 |
| PIWA | 0.0000 | 0.0000 | 0.0834 | 0.0139 | 0.0000 | 0.0278 | 0.0000 | 14.5833 |
| RBWO | 0.0100 | 0.0000 | 0.0000 | 0.0000 | 0.0000 | 0.0000 | 0.0000 | 14.6083 |
| RCKI | 0.0000 | 0.0000 | 0.1313 | 0.0000 | 0.0000 | 0.3063 | 0.0063 | 8.3312 |
| TUTI | 0.0000 | 0.0179 | 0.0536 | 0.0000 | 0.0000 | 0.0357 | 0.0000 | 11.1111 |
| WEVI | 0.0000 | 0.0000 | 0.1333 | 0.0000 | 0.0667 | 0.0333 | 0.1000 | 8.2500 |
| YBSA | 0.0000 | 0.0000 | 0.0000 | 0.0000 | 0.0000 | 0.0000 | 0.0000 | 15.8298 |
| YTWA | 0.0000 | 0.0145 | 0.0435 | 0.0580 | 0.0000 | 0.0000 | 0.0000 | 15.2246 |

| **Species** | **N total individuals** | **N all maneuvers** | **Mean foraging height** | **Mean foliage density** | **Most freq. substrate** | **Most freq. maneuver** | **Most freq. distance** |
| --- | --- | --- | --- | --- | --- | --- | --- |
| AMGO | 3 | 10 | 21.31 | 1.75 | Fruiting Body | Probe | Far |
| BAWW | 25 | 163 | 10.78 | 1.29 | Trunk | Glean | Near |
| BGGN | 27 | 102 | 12.55 | 2.28 | Live Leaf | Glean | Far |
| BHVI | 22 | 42 | 11.23 | 1.50 | Branch | Glean | Medium/Near |
| CACH | 13 | 42 | 12.18 | 2.46 | Branch | Glean | Far |
| CAWR | 6 | 18 | 4.17 | 2.61 | Dead Leaf | Probe | Near |
| DOWO | 12 | 61 | 16.05 | 1.18 | Trunk | Hammer | Near |
| EAPH | 13 | 32 | 9.63 | 1.55 | Branch | Sally | Far |
| ETTI | 17 | 56 | 11.11 | 2.60 | Branch | Glean/Probe | Far |
| MYWA | 19 | 71 | 9.92 | 2.26 | Branch | Glean | Near |
| NOCA | 13 | 47 | 4.28 | 1.53 | Ground | Reach-down | Far |
| OCWA | 12 | 63 | 9.65 | 2.27 | Dead Leaf | Probe | Far |
| OVEN | 5 | 26 | 0.00 | 2.27 | Ground | Reach | - |
| PIWA | 19 | 72 | 14.58 | 2.03 | Epiphyte | Probe | Far |
| RBWO | 24 | 100 | 14.61 | 1.42 | Trunk | Probe | Near |
| RCKI | 39 | 159 | 8.32 | 2.48 | Live Leaf | Glean | Far |
| WEVI | 11 | 27 | 7.91 | 2.74 | Live Leaf | Glean | Far |
| YBSA | 7 | 37 | 15.83 | 0.81 | Trunk | Hammer | Near |
| YTWA | 18 | 69 | 15.22 | 2.29 | Dead Leaf | Probe | Far |

**Table S8. Summarized foraging data.** These data were collected in the same habitats and during the same time of year on species that were tested in the playback trials but collected independently from playback trials. Species codes correspond to four letter Alpha Codes, as established by the American Ornithologists’ Union (see Table 9, Supplement S1). Sample sizes represent number of independent individuals observed foraging and total number of observed foraging maneuvers respectively. Foraging height was estimated to the nearest meter for each foraging maneuver with the help of a rangefinder. Distance from tree trunk was binned into three categories (near to trunk, medium, far from trunk) by dividing the branch on which a focal individual was perched into thirds. Foliage density is calculated based on percentage of light penetrating the foliage on a 0 to 5 scale in a 1 m diameter sphere around the focal individual, and substrates and attack maneuvers were categorized following Remsen and Robinson (1990). Most frequent substrate and maneuver were those observed at the highest frequency across all foraging observations.

| **Alpha Code** | **Species Name** | **Latin Name** | **Family** |
| --- | --- | --- | --- |
| ACFL | Acadian Flycatcher | *Empidonax virescens* | Tyrannidae |
| AMGO | American Goldfinch | *Spinus tristis* | Fringillidae |
| AMRE | American Redstart | *Setophaga ruticilla* | Parulidae |
| AMRO | American Robin | *Turdus migratorius* | Turdidae |
| BAWW | Black-and-white Warbler | *Mniotilta varia* | Parulidae |
| BGGN | Blue-gray Gnatcatcher | *Polioptila caerulea* | Polioptilidae |
| BHVI | Blue-headed Vireo | *Vireo solitarius* | Vireonidae |
| BLJA | Blue Jay | *Cyanocitta cristata* | Corvidae |
| BTBW | Black-throated Blue Warbler | *Setophaga caerulescens* | Parulidae |
| CACH | Carolina Chickadee | *Poecilotis carolinensis* | Paridae |
| CAWR | Carolina Wren | *Thyothorus ludovicianus* | Troglodytidae |
| CHSP | Chipping Sparrow | *Spizella passerina* | Passerelidae |
| DOWO | Downy Woodpecker | *Dryobates pubescens* | Picidae |
| EAPH | Eastern Phoebe | *Sayornis phoebe* | Tyrannidae |
| EAWP | Eastern Wood-pewee | *Contopus virens* | Tyrannidae |
| GRCA | Gray Catbird | *Dumetella carolinensis* | Mimidae |
| HETH | Hermit Thrush | *Catharus guttatus* | Turdidae |
| MAWA | Magnolia Warbler | *Setophaga magnolia* | Parulidae |
| MYWA | "Myrtle" Yellow-rumped Warbler | *Setophaga coronata coronata* | Parulidae |
| NOCA | Northern Cardinal | *Cardinalis cardinalis* | Cardinalidae |
| OCWA | Orange-crowned Warbler | *Oreothlypis celata* | Parulidae |
| OVEN | Ovenbird | *Seiurus aurocapilla* | Parulidae |
| PAWA | Palm Warbler | *Setophaga palmarum* | Parulidae |
| PIWA | Pine Warbler | *Setophaga pinus* | Parulidae |
| RBWO | Red-bellied Woodpecker | *Melanerpes carolinensis* | Picidae |
| RCKI | Ruby-crowned Kinglet | *Regulus calendula* | Regulidae |
| TUTI | Tufted Titmouse | *Baeolophus bicolor* | Paridae |
| WEVI | White-eyed Vireo | *Vireo griseus* | Vireonidae |
| WOTH | Wood Thrush | *Hylocichla mustelina* | Turdidae |
| YBCU | Yellow-billed Cuckoo | *Coccyzus americanus* | Cuculidae |
| YBSA | Yellow-bellied Sapsucker | *Sphyrapicus varius* | Picidae |
| YTWA | Yellow-throated Warbler | *Setophaga dominica* | Parulidae |

**Table S9. Species names and Alpha Codes of all species included in the study.**

**Table S10. Frequency of use of five Z call exemplars by species.** Playback exemplars represent five unique recordings of Tufted Titmouse alarm calls made into 30-second playback stimulus tapes and presented to free-living birds. Playback exemplar was only recorded during the second winter of playback presentations (N = 125 playback trials out of 242), sample sizes of playbacks for which the exemplar was not recorded are listed as ‘not recorded’. N refers to the total number of playback presentations made to each species. Full species names are listed in Table 9, Supplement S1.

| Species | Playback Exemplar | | | | | Not Recorded | N |
| --- | --- | --- | --- | --- | --- | --- | --- |
|  | 1 | 2 | 3 | 4 | 5 |  |  |
| ACFL | 0 | 0 | 1 | 0 | 0 | 0 | 1 |
| AMGO | 0 | 0 | 1 | 1 | 1 | 2 | 5 |
| AMRE | 1 | 1 | 0 | 1 | 1 | 1 | 4 |
| AMRO | 1 | 0 | 0 | 0 | 0 | 0 | 1 |
| BAWW | 1 | 1 | 3 | 1 | 0 | 10 | 16 |
| BGGN | 0 | 1 | 2 | 1 | 1 | 9 | 14 |
| BHVI | 0 | 2 | 2 | 0 | 0 | 10 | 14 |
| BLJA | 3 | 0 | 1 | 0 | 0 | 0 | 4 |
| BTBA | 0 | 1 | 0 | 1 | 0 | 0 | 2 |
| CACH | 0 | 2 | 2 | 1 | 0 | 5 | 10 |
| CAWR | 2 | 1 | 2 | 1 | 1 | 1 | 8 |
| CHSP | 0 | 0 | 0 | 0 | 0 | 2 | 2 |
| DOWO | 1 | 1 | 3 | 0 | 3 | 2 | 9 |
| EAPH | 1 | 0 | 1 | 1 | 1 | 12 | 16 |
| EAWP | 0 | 1 | 1 | 0 | 0 | 1 | 3 |
| GRCA | 0 | 0 | 1 | 0 | 0 | 1 | 2 |
| HETH | 2 | 0 | 2 | 2 | 2 | 2 | 10 |
| MAWA | 0 | 0 | 1 | 0 | 0 | 0 | 1 |
| MYWA | 0 | 0 | 2 | 0 | 1 | 10 | 13 |
| NOCA | 4 | 1 | 0 | 0 | 3 | 10 | 18 |
| OCWA | 1 | 0 | 5 | 0 | 0 | 5 | 11 |
| OVEN | 3 | 0 | 0 | 0 | 1 | 2 | 6 |
| PIWA | 1 | 1 | 1 | 5 | 0 | 1 | 9 |
| RBWO | 2 | 2 | 1 | 1 | 2 | 3 | 11 |
| RCKI | 1 | 1 | 4 | 1 | 1 | 11 | 19 |
| WEVI | 1 | 0 | 3 | 0 | 2 | 4 | 10 |
| WOTH | 1 | 0 | 0 | 0 | 0 | 0 | 1 |
| YBCU | 0 | 0 | 1 | 0 | 0 | 0 | 1 |
| YBSA | 1 | 1 | 1 | 0 | 2 | 5 | 10 |
| YPWA | 0 | 0 | 0 | 0 | 0 | 1 | 1 |
| YTWA | 1 | 1 | 0 | 1 | 0 | 7 | 10 |
|  | **28** | **18** | **41** | **18** | **22** | **117** | **242** |

**Table S11. Playback response as a function of playback exemplar.** Playback response refers to the proportion of times that an individual responded to the Z call stimulus for each combination of species and exemplar. Sample sizes for the number of playbacks of each exemplar can be found in Table 11. Only species with four or more playback presentations that were included in the GLM analysis are listed in the table (other species presented with playback are listed in Table 1). N refers to the total number of playback presentations made to each species. Full species names are listed in Table 9, Supplement S1.

| **Species** | **Response proportion by exemplar** | | | | | **N** |
| --- | --- | --- | --- | --- | --- | --- |
|  | **1** | **2** | **3** | **4** | **5** |  |
| AMGO |  |  | 1.00 | 1.00 | 1.00 | 5 |
| AMRE |  | 1.00 |  | 1.00 | 1.00 | 4 |
| BAWW | 1.00 | 1.00 | 1.00 | 1.00 |  | 16 |
| BGGN |  | 1.00 | 1.00 | 1.00 | 1.00 | 14 |
| BHVI |  | 1.00 | 1.00 |  |  | 14 |
| BLJA | 0.66 |  | 1.00 |  |  | 4 |
| CACH |  | 1.00 | 1.00 | 1.00 |  | 10 |
| CAWR | 0.50 | 1.00 | 1.00 | 1.00 | 1.00 | 8 |
| DOWO | 1.00 | 1.00 | 1.00 |  | 1.00 | 9 |
| EAPH | 0.00 |  | 0.00 | 0.00 | 0.00 | 16 |
| HETH | 1.00 |  | 1.00 | 1.00 | 1.00 | 10 |
| MYWA |  |  | 1.00 |  | 1.00 | 13 |
| NOCA | 1.00 | 1.00 |  |  | 1.00 | 18 |
| OCWA | 1.00 |  | 1.00 |  |  | 11 |
| OVEN | 1.00 |  |  |  | 1.00 | 6 |
| PIWA | 1.00 | 1.00 | 1.00 | 1.00 |  | 9 |
| RBWO | 0.50 | 1.00 | 1.00 | 1.00 | 1.00 | 11 |
| RCKI | 1.00 | 1.00 | 1.00 | 1.00 | 1.00 | 19 |
| WEVI | 1.00 |  | 1.00 |  | 1.00 | 10 |
| YBSA | 1.00 | 1.00 | 1.00 |  | 1.00 | 10 |
| YTWA | 1.00 | 1.00 | 0.00 | 1.00 | 0.00 | 10 |

**Table S12. Mean and standard deviations of maximum dBA of playback exemplars measured at 1 meter.** Exemplars are 30-second recordings of the titmouse Z call used during playback presentations to free-living birds and 30-second recordings of frogs used during the control playback. Amplitudes were measured *post hoc* at a 1-meter distance in the same habitat type as the experimental playbacks, using the same standardized speaker volume as that used in the field and the same speaker type. Amplitude was measured as A-weighted decibels using a digital sound meter on fast setting. Amplitude measurements represent the average of five maximum dBA measurements. Overall mean and standard deviation are given in bold at the bottom of the table.

| **Exemplar** | **Amplitude (dBA)** | **SD** |
| --- | --- | --- |
| **Experimental stimulus** | | |
| 1 | 76.72 | 1.53 |
| 2 | 75.88 | 1.73 |
| 3 | 74.98 | 1.97 |
| 4 | 75.88 | 0.73 |
| 5 | 75.54 | 0.89 |
|  | **75.80** | **1.37** |
| **Control (frog) playback** | | |
| 1 | 78.04 | 1.29 |
| 2 | 81.64 | 1.74 |
| 3 | 80.80 | 0.97 |
|  | **80.16** | **1.33** |
