# Supplementary material for: Foraging ecology drives social information reliance in an avian eavesdropping community
Source: Ecol Evol. 2019 Sep 14;9(20):11584–97. doi: 10.1002/ece3.5561 (PMC6822049; doi:10.1002/ece3.5561)
Supplement: Supplementary file 2 [file ECE3-9-11584-s002.docx]

**Appendix S2: Principal Coordinate Analysis Methods and Interpretation**

**METHODS**

**Data reduction of foraging and microhabitat variables**

All statistical analyses were performed in R (version 3.5.1). We first aggregated all of the foraging observation data (37 variables for each species; Table 3, Supplement S2) for species with more than five independent foraging observations and determined the proportions of each foraging maneuver, distance category from trunk, foliage density category, and foraging substrate observed for each species (Tables 5-7, Supplement S1). We removed single observations of a foraging maneuver or substrate for any given species to avoid biasing subsequent analyses with outliers, and combined all types of reach and hang maneuvers respectively. We then ordinated the foraging data at the species level to describe covariance patterns and as a variable reduction technique. We used the Gower dissimilarity index (Gower 1971) to create a dissimilarity matrix, using the *daisy* function in the *cluster* package, which we then analyzed using Principal Coordinates Analysis (PCoA; Gower 2015) using the *cmdscale* function in the *stats* package. We selected PCoA because our data do not fit the assumptions of Principal Components Analysis (lack of multivariate normality, mixed categorical and continuous predictors, more predictor variables than samples) and the relaxed assumptions of this technique allow for unconstrained ordination of such data sets (McGarigal, Cushman & Stafford 2000). We interpreted the PCoA axes by projecting the weighted averages of the scores for each predictor variable onto a biplot using the *wascores* function (*vegan* package). We also performed PCoA on the microhabitat variables (5 variables per playback; Table 4, Supplement S2) recorded during playback to reduce the number of predictor variables for our final models. Because these data contained a mix of categorical and continuous predictor variables, they could not be projected onto the biplot. We therefore interpreted these axes using the averages of the predictor variables for extreme values of each principal coordinate axis (Figure 1, Supplement S2; Table 4, Supplement S2). We selected important axes to retain for further analyses by consulting a scree plot, and by retaining only interpretable axes.

**RESULTS**

**Interpreting PCoA axes: playback trial microhabitat**

We selected the first three coordinate axes of the analysis of the microhabitat variables (Table 1, Supplement S2), accounting for 77% of the observed variance, based on analysis of a scree plot. We interpret the first axis (36% of variance, Edge-MH) as a measure of forest edge versus forest interior sites. Based on an examination of extreme values (Figure 1; Table 4, Supplement S2), higher values were associated with sites within 50 m of a hard edge, further distances from the trunk, lower heights from ground, higher vegetation density, and lower distance to cover, while lower values were associated with forest interior sites with lower vegetation density, greater distance to cover, and greater height from the ground (Table 4, Supplement S2). The second axis (25% of variance, Trunk-MH) represents a measure of distance from trunk in terms of microhabitat. Large values represent sites that are nearer to the trunk, with low vegetation density and greater distances to cover, while smaller values represent sites farther from the trunk, with higher vegetation density and smaller distances to cover (Table 4, Supplement S2). The third axis (16% of variance, Escape-MH) represents a measure of availability of escape cover. Large values had high distance to cover, low vegetation density, greater height from ground, and either were located on the trunk or in exposed sites far from the trunk. Smaller values had low distance to cover and tended to be located at lower heights in dense vegetation at intermediate distances from the trunk (Table 4, Supplement S2).

**Interpreting PCoA axes: species-level winter foraging niche**

Based on examination of a scree plot and axis interpretability, we selected the first four coordinate axes to use as predictor variables for later analyses. These four coordinate axes describe 68% of variation in the foraging data among them (Table 2, Supplement S2). The first coordinate axis (hereafter Trunk-F) describes 26% of the variation in the foraging data. Based on the factor loadings (Table 3, Supplement S2), we interpret this axis as a measure of distance of the species’ foraging niche from the trunk. Higher values were associated with foraging nearer to the trunk, use of the trunk as a substrate, and trunk-based foraging maneuvers such as hammer and peck (Figure 2a; Table 3, Supplement S2). Lower values were associated with more frequent use of microhabitats further from the trunk, the use of foliage or air as a foraging substrate, and aerial foraging maneuvers (flush-pursue, sally hover). The second axis (Occlusion-F) explains 17% of the variance and represents a measure of the visual occlusion associated with the foraging maneuvers of each species. Higher values were associated with substrates that occlude vision (dead leaves, epiphytes) and probing foraging maneuvers that limit vigilance, while lower values were associated with more frequent use of open sites, such as tree trunks or capturing prey items in the air, as well as aerial or trunk-based foraging maneuvers (sally, hammer; Table 3). Species with high values on this coordinate axis consisted of an assemblage of epiphyte-probing birds including Orange-crowned Warbler (*Oreothlypis celata*), Pine Warbler (*Setophaga pinus*), and Yellow-throated Warbler (*Setophaga dominica*).

The third foraging axis (Height-F) explains 14% of the variance in the foraging dataset, and is a measure of the foraging height of each species. Higher values on this principal coordinate axis were associated with lower foraging heights and increased use of the ground as a foraging substrate, while negative values were associated with greater foraging heights and canopy substrates such as branches, pine needles, and pine cones (Figure 2b; Table 3). Ground-foraging species such as Northern Cardinal (*Cardinalis cardinalis*) and Ovenbird (*Seiurus aurocapillus*) had high values on this coordinate axis. The fourth axis (Aerial-F) explains 10% of the total variance and was more complicated to interpret. Lower values were associated with increased use of microhabitats with high vegetation density (4 on the scale used here), and increased use of substrates associated with dense vegetation (vine tangles, dead leaf clusters). In contrast, higher values were associated with greater use of more open habitats and aerial foraging maneuvers (sallies; Table 3). As such, we chose to interpret this axis as a measure of degree of aerial foraging behavior for a species. High values on this axis were associated with a sit-and-wait aerial flycatcher (Eastern Phoebe, *Sayornis phoebe*).

**LITERATURE CITED**

Gower, J. (2015) Principal Coordinates Analysis. *Wiley StatsRef: Statistics Reference Online,* **1**.

Gower, J.C. (1971) A general coefficient of similarity and some of its properties. *Biometrics,* **27,** 857-871.

McGarigal, K., Cushman, S. & Stafford, S. (2000) *Multivariate statistics for wildlife and ecology research*. Springer-Verlag, York, PA.

**TABLES**

**Table S1. Principal coordinate axes of the microhabitat measures collected before playback.** Principal coordinate axes were obtained by ordinating 6 microhabitat variables collected at the location of the focal individual before playback (Table 4). We retained the first three axes (named) for further analyses based on cumulative variance explained and consultation of a scree plot (McGarigal et al. 2000).

| **Coordinate axis** | **Eigenvalue** | **Proportion of variance explained** | **Cumulative variance** |
| --- | --- | --- | --- |
| Edge-MH | 6.036 | 0.359 | 0.359 |
| Trunk-MH | 4.199 | 0.249 | 0.608 |
| Escape-MH | 2.745 | 0.163 | 0.771 |
| 4 | 2.385 | 0.142 | 0.913 |
| 5 | 1.467 | 0.087 | 1.000 |

**Table S2. Principal coordinate axes from analysis of the foraging ecology data.** We obtained 16 principal coordinate axes by ordinating 37 foraging variables (Table 3) collected from field observations of foraging birds. We retained the first four axes (named) for further analyses based on cumulative variance explained, interpretability, and consultation of a scree plot.

| **Coordinate axis** | **Eigenvalue** | **Proportion of variance explained** | **Cumulative variance** |
| --- | --- | --- | --- |
| Trunk-F | 0.152 | 0.264 | 0.264 |
| Occlusion-F | 0.098 | 0.171 | 0.434 |
| Height-F | 0.085 | 0.147 | 0.582 |
| Aerial-F | 0.059 | 0.102 | 0.684 |
| 5 | 0.046 | 0.079 | 0.764 |
| 6 | 0.029 | 0.051 | 0.814 |
| 7 | 0.023 | 0.040 | 0.855 |
| 8 | 0.021 | 0.037 | 0.891 |
| 9 | 0.019 | 0.033 | 0.925 |
| 10 | 0.015 | 0.026 | 0.950 |
| 11 | 0.010 | 0.017 | 0.967 |
| 12 | 0.008 | 0.014 | 0.981 |
| 13 | 0.005 | 0.009 | 0.990 |
| 14 | 0.004 | 0.007 | 0.996 |
| 15 | 0.002 | 0.003 | 1.000 |
| 16 | 0.000 | 0.000 | 1.000 |

**Table S3. Factor loadings for the foraging ecology principal coordinates used in the analysis.** Variables shown consist of 37 measures of foraging behavior and microhabitat obtained from foraging observations of free-living birds. Named principal coordinate axes are, from left to right, the first four axes, explaining 68% of variance (Table 2).

| **Category** | **Variable** | **Trunk-F** | **Occlusion-F** | **Height-F** | **Aerial-F** |
| --- | --- | --- | --- | --- | --- |
| Foraging Substrate | Air | -0.087 | -0.054 | -0.004 | 0.036 |
|  | Branch | -0.026 | -0.027 | -0.014 | -0.004 |
|  | Dead Branch | 0.072 | -0.016 | -0.047 | -0.012 |
|  | Dead Leaf | -0.018 | 0.088 | -0.033 | -0.018 |
|  | Epiphyte | -0.016 | 0.028 | -0.048 | -0.013 |
|  | Fruiting Body | 0.080 | 0.085 | 0.015 | 0.114 |
|  | Ground | 0.013 | 0.031 | 0.157 | 0.004 |
|  | Live Leaf | -0.086 | -0.017 | 0.005 | -0.002 |
|  | Pine Cone | -0.015 | 0.102 | -0.073 | -0.019 |
|  | Pine Needles | -0.039 | 0.041 | -0.054 | 0.024 |
|  | Trunk | 0.099 | -0.055 | -0.011 | -0.016 |
|  | Vine | -0.005 | -0.031 | -0.010 | -0.043 |
| Distance from Trunk | Far | -0.028 | 0.009 | -0.007 | 0.016 |
|  | Medium | -0.012 | -0.011 | -0.016 | -0.002 |
|  | Near | 0.032 | -0.012 | -0.013 | -0.015 |
| Vegetation Density at Foraging Site | 0 | 0.024 | -0.048 | 0.036 | 0.004 |
|  | 1 | 0.047 | -0.019 | -0.007 | 0.012 |
|  | 2 | -0.009 | 0.013 | 0.001 | 0.006 |
|  | 3 | -0.048 | 0.014 | -0.009 | -0.011 |
|  | 4 | -0.011 | 0.020 | 0.033 | -0.038 |
| Foraging Maneuver | Flake | 0.047 | 0.012 | -0.059 | -0.018 |
|  | Flush-pursue | -0.089 | -0.032 | -0.017 | 0.007 |
|  | Gape | -0.019 | 0.145 | -0.057 | 0.037 |
|  | Glean | -0.035 | -0.017 | -0.002 | -0.016 |
|  | Hammer | 0.127 | -0.055 | -0.014 | -0.024 |
|  | Hang | -0.041 | 0.009 | -0.025 | -0.030 |
|  | Hang-down Probe | 0.048 | 0.094 | -0.014 | 0.078 |
|  | Lunge | 0.028 | 0.048 | 0.181 | -0.003 |
|  | Peck | 0.151 | -0.046 | -0.066 | 0.013 |
|  | Probe | 0.041 | 0.061 | -0.028 | 0.012 |
|  | Reach Down | -0.005 | 0.024 | 0.117 | -0.004 |
|  | Reach-down Probe | -0.018 | 0.128 | -0.063 | 0.018 |
|  | Reach-up | -0.062 | -0.003 | 0.001 | -0.010 |
|  | Sally | -0.086 | -0.079 | 0.010 | 0.073 |
|  | Sally-hover | -0.083 | -0.058 | -0.007 | 0.025 |
|  | Sally-pounce | -0.074 | -0.078 | -0.009 | 0.022 |
| Foraging Height | Average Foraging Height | 0.015 | -0.002 | -0.019 | 0.009 |

**Table S4. Average values for large and small principal coordinate scores of microhabitat data.** Principal coordinate axes correspond to the first three axes and where those retained for analyses (Table 1, Supplement S2). We interpreted the principal coordinate axes by subsetting all data-points into extreme (small and large) values (Figure 1, Supplement S2). Values given are averages of continuous variables or counts in the case of categorical variables for the subset. See results section for interpretation.

| **Coordinate axis** | **Subset** | ***N*** | **Mean Distance from Speaker** | **Mean Height from Ground** | **# Far from Trunk** | **# Medium from Trunk** | **# Near trunk** | **Mean Vegetation Density** | **Mean Distance to Cover** | **Number of Edge Sites** | **Number of Interior Sites** |
| --- | --- | --- | --- | --- | --- | --- | --- | --- | --- | --- | --- |
| Edge-MH | Small values | 19 | 18.89 | 21.89 | 0 | 1 | 17 | 1.263 | 9 | 0 | 19 |
|  | Large values | 52 | 15.00 | 8.49 | 26 | 18 | 1 | 2.385 | 2.853 | 52 | 0 |
| Trunk-MH | Small values | 24 | 20.09 | 15.22 | 24 | 0 | 0 | 2.833 | 1.292 | 3 | 21 |
|  | Large values | 41 | 18.23 | 12.82 | 0 | 0 | 40 | 1.439 | 5.305 | 30 | 11 |
| Escape-MH | Small values | 44 | 14.70 | 9.05 | 0 | 36 | 4 | 2.795 | 1.655 | 18 | 26 |
|  | Large values | 38 | 21.39 | 15.93 | 32 | 0 | 5 | 1.368 | 5.263 | 16 | 22 |

**FIGURES**


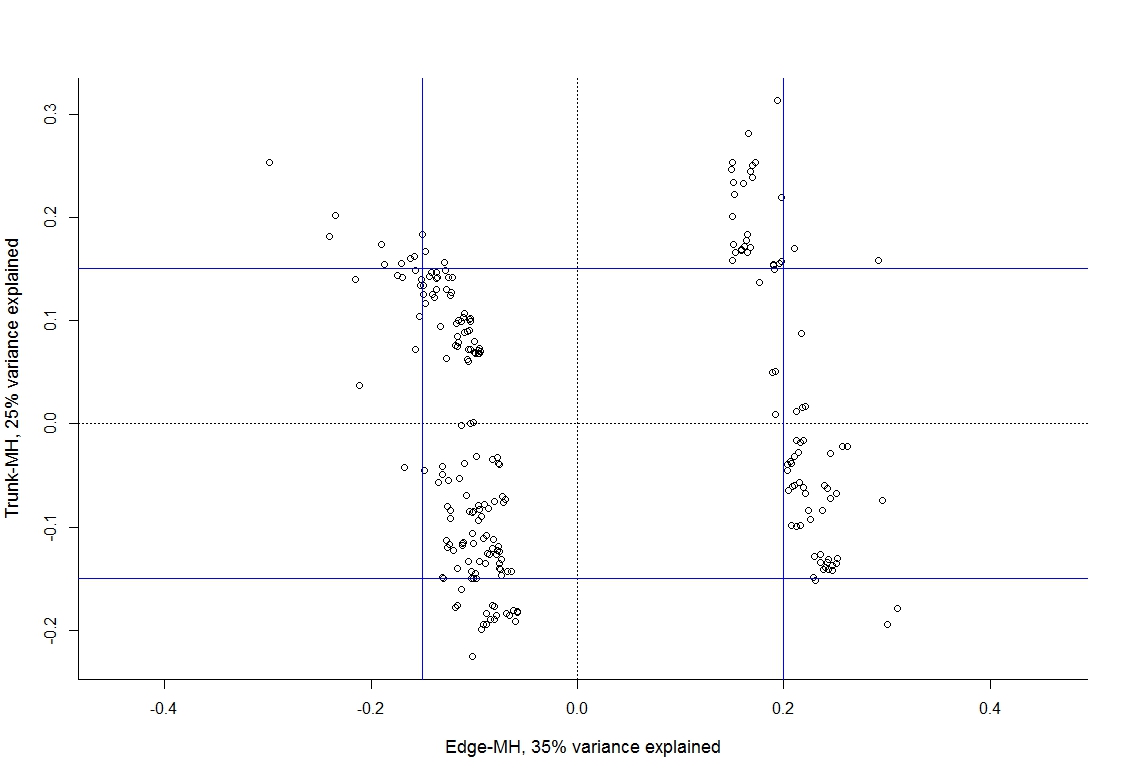


**Figure S1. Biplot of principal coordinate axes Edge-MH and Trunk-MH of the microhabitat variables.** These axes represent the first and second axes obtained from principal coordinates analysis of 6 microhabitat variables (see Table 4, Supplement S2) recorded before each Z call playback. Points represent playback locations (samples) while blue lines indicate cut-off values used to define large and small sub-samples from which average values in Table 4, Supplement S2 were calculated.

**
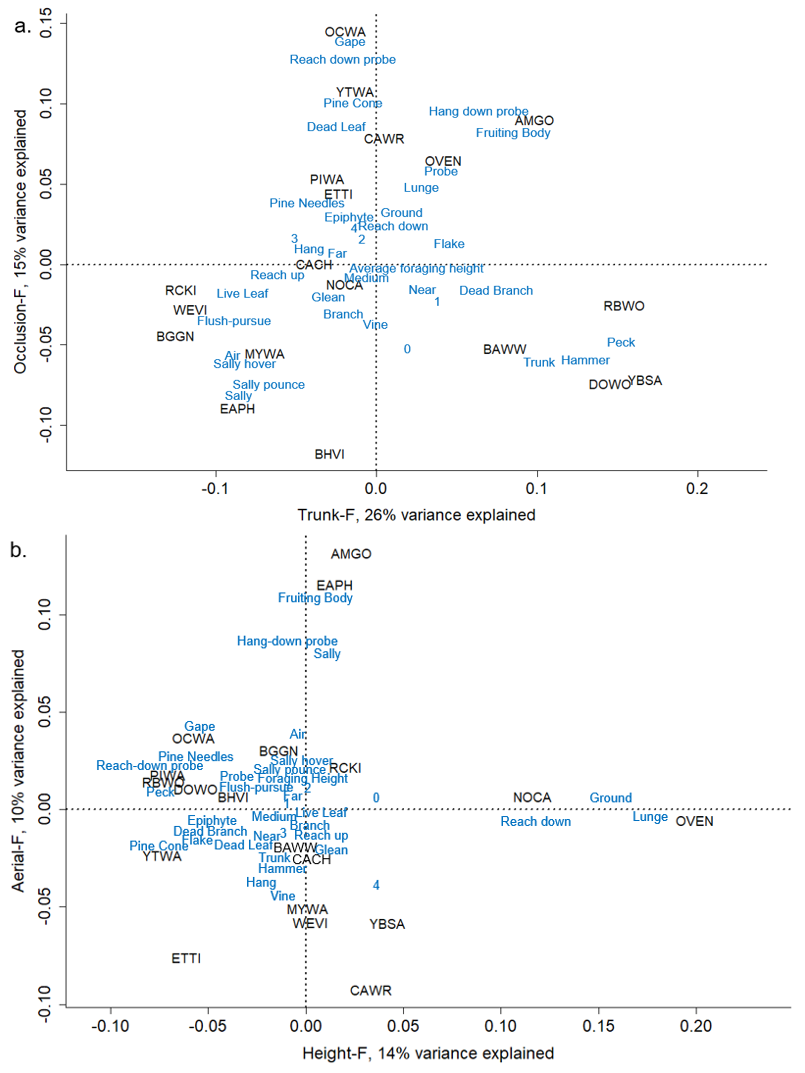
**

**Figure S2. (A) Biplot of Trunk-F and Occlusion-F.** These represent the first and second principal coordinate axes derived from 37 predictor variables collected during foraging observations (Table 3, Supplement S2), representing 43% of the variance (Table 2, Supplement S2). Blue labels represent weighted averages of predictor variables across all samples and black labels represent the coordinate scores for each bird species. Species names are abbreviated using four-letter Alpha Codes. Axis interpretation is described in detail in the results section. **(B) Biplot of Height-F and Aerial-F.** These represent the third and fourth principal coordinates, representing 25% of the variance (Table 2, Supplement S2). Height-F is a measure of the foraging height of each species.
